# Efficient synthesis of 1,3-naphtoxazine derivatives using reusable magnetic catalyst (GO-Fe_3_O_4_–Ti^(IV)^): anticonvulsant evaluation and computational studies

**DOI:** 10.1186/s13065-022-00836-8

**Published:** 2022-06-10

**Authors:** Soghra Khabnadideh, Aida solhjoo, Reza Heidari, Leila Amiri Zirtol, Amirhossein Sakhteman, Zahra Rezaei, Elaheh Babaei, Samaneh Rahimi, Leila Emami

**Affiliations:** 1 Pharmaceutical Sciences Research Center, Shiraz University of Medical Sciences, Shiraz, Iran; 2 Department of Medicinal Chemistry, Faculty of Pharmacy, Shiraz University of Medical Sciences, Shiraz, Iran; 3 Department of Chemistry, College of Science, Yazd University, Yazd, Iran

**Keywords:** GO-Fe_3_O_4_–Ti^(IV)^, Catalyst, 1,3-Oxazine, Anticonvulsant, PTZ, Molecular simulation

## Abstract

A series of 2-aryl/alkyl-2,3-dihydro-1*H*-naphtho[1,2-*e*][1,3]oxazines ***(S***_***1***_***–S***_***11***_***)*** were synthesized with an eco-friendly and recoverable nanocatalyst (GO-Fe_3_O_4_–Ti^(IV)^) as an efficient magnetic composite. The new nanocatalyst was characterized by FT-IR, XRD and, EDS analysis. A conformable procedure, easy to work up and having a short reaction time with high yields are some advantages of this method. The new catalyst is also thermal-stable, reusable and, environment-friendly. The chemical structures of the synthesized 1,3-oxazine compounds were confirmed by comparing their melting points with those reported in literature. Then, the anticonvulsant activity of these compounds was assessed by the intraperitoneal pentylenetetrazole test (ipPTZ). Compounds ***S***_***10***_ and ***S***_***11***_ displayed considerable activity against chemically-induced seizure tests. The molecular simulation was also done to achieve their binding affinities as γ‐aminobutyric acid A (GABA‐A) receptor agonists as an assumptive mechanism of their anticonvulsant action. The result of molecular studies represented strongly matched with biological activity. Molecular docking simulation of the potent compound *(****S***_***10***_*)* and diazepam as the positive control was performed and some critical residues like Thr262, Asn265, Met286, Phe289, and Val290 were identified. Based on the anticonvulsant results and also in silico ADME predictions, ***S***_***11***_ can be to become a potential drug candidate as an anticonvulsant agent.

**Supplementary Information:**

The online version contains supplementary material available at 10.1186/s13065-022-00836-8.

## Introduction

Epilepsy is one of the most common chronic neurological syndromes responsible for substantial morbidity and mortality [1, 2]. Many currently available antiepileptic drugs have shown a lack of efficacy, high rates of adverse effects, and drug-drug interactions. So, many patients are not thoroughly pleased with the available drugs [3, 4]. Epilepsy dose not well controlled in over 30% of patients with the available medications [5]. The existing drugs showed dose-related toxicity and idiosyncratic side effects. Therefore, search for anti-epileptic agents with lower toxicity and higher selectivity remains as a motivating topic in medicinal chemistry [6].

Recent research represented that several oxazine derivatives could be as potential novel candidates in drug discovery. Some properties including, versatility of the oxazine skeleton, relative chemical simplicity and accessibility, retained these chemicals among the most promising sources of bioactive compounds [7]. Oxazine analogues were found to represent appropriate pharmacological effects such as anti-bacterial, anti-coagulant, cytotoxic, analgesic, antipyretic anticonvulsant, and anti-tumor [8–12]. Oxazine with naphthalene rings, called naphthoxazines have different pharmacological activities such as antiparkinson, psycho stimulating, anti-tumor, and antidepressant [10]. Besides, this structure can be used as potent forerunners in organic synthesis to found novel biologically active molecules [13]. Hence, these compounds are an interesting category of chemical for researchers, and there is enough scope to explore new oxazine derivatives. So, many researchers have been considered to facile the synthesizing of such fundamental heterocyclic rings [8]. Several procedures for the preparation of oxazine analogous have been previously described [10, 11, 14]. A large library of these compounds is obtained by Betti's reaction between activated phenols, amines and aryl aldehydes [15, 16]. In the desired procedure, 1,3-oxazines were prepared by amidoalkyl naphthols, which were synthesized by Lewis or Brønsted acid catalysts such as *p*-toluenesulfonic acid, H_2_NSO_3_H, oxalic acid, and CPTS [11]. A series of some 1, 3-oxazine amine derivatives have also been synthesized by solid SiO_2_-H_3_PO_4_ catalyzed solvent-free cyclization under microwave irradiation [17]. The synthesis of dihydro furonaphthoxazine derivatives was likewise performed through a Mannich-type condensation [18]. However, some of the reported methods suffer from disadvantages including long reaction time, harsh reaction conditions [11], and few of them have been focused on the multicomponent reactions method [14]. Therefore, to overcome these limitations, the discovery of a new, easily available catalyst with high catalytic activity for the preparation of oxazine is still desirable [11]. In this regard, acid catalysts were used as appropriate catalysts to synthesize of heterocyclic compounds. Dextrin, graphene oxide and cellulose are different substrates that used to produce heterogeneous acid catalysts. Magnetic catalyst is suitable and green procedure for synthesize of organic compounds [19–22]. In our previous work we introduced an effective and reusable heterogeneous catalyst with immobilize Ti on magnetized graphene oxide (GO-Fe_3_O_4_–Ti^(IV)^) to synthesize bis (indolyl) methanes and benzo[a]xanthen-11-one [23]. Here we would like to report the synthesis of some 1,3-naphtoxazine derivatives using 2-naphthol, aromatic amines and formaldehyde in the presence of this heterogeneous acid catalyst through one-pot reactions under solvent-free conditions. The present method is superior to previous reports due to its solvent-free condition. The prepared compounds were examined for their anticonvulsant activity by intraperitoneal pentylenetetrazole test (ipPTZ). As anticonvulsant activity in many seizure models is achieved by increasing γ-aminobutyric acid (GABA) transmission therefore, a presumptive mechanism for anticonvulsant activity was described as γ‐aminobutyric acid (GABA) agonist. For this purpose, these compounds were applied to molecular docking simulation to find out their binding conformations and structural specificities towards the GABA agonist as plausible targets in convulsion management. In silico physicochemical parameters, and ADME (Absorption, Distribution, Metabolism and Excretion) profiling calculations also were done to achieve pharmacokinetic properties of the 1,3-naphtoxazine compounds.

## Experimental section

### Chemistry

All chemicals, solvents, and reagents were purchased from Sigma and Merck and used without any purification. The products were characterized by FT-IR, ^1^H NMR, and ^13^C-NMR. FT-IR spectra were run on Bruker Equinox spectrometer. The ^1^HNMR and ^13^C-NMR were recorded by a Bruker (DRX-400 Avance). Melting points were determined by a Buchi melting point B-540 B.V.CHI apparatus and comprised of those reported in the literature [6]. Pentylenetetrazole (PTZ) was dissolved in a physiological saline solution and DMSO was used as co-solvent. Diazepam was purchased from Kimidaroo (Iran) and was dissolved in normal saline. In all experiments, PTZ was administered intravenously (iv) and all other compounds were administered intraperitoneally (i.p.) 30 min before PTZ.

### General procedure for the preparation of GO-Fe_3_O_4_-Ti^(IV)^

To prepare the GO-Fe_3_O_4_-Ti^(IV)^, the pre-synthesized GO (0.3 g) via the Hummers method [24] was dispersed in 25 mL water (30 min) at room temperature and then 0.8 gr of FeCl_3_.6H_2_O and 0.3 gr of FeCl_2_.4H_2_O were added dropwise to the solution. The temperature increased to 85 °C, and a 30% aqueous ammonia solution was added to raise the pH to 10. Afterward, the solution was stirred for 45 min and cooled to room temperature. Instantly, the resulting black precipitate was centrifuged, washed three times with deionized (DI) water, and dried at 60 °C for 24 h. Then, 0.5 g of prepared GO-Fe_3_O_4_ was added to 5 mL of dry CHCl_3_ at room temperature under violent stirring for 30 min. Subsequently, 2 mL of TiCl_4_ was added slowly and stirred at room temperature for 1 h. The resulting GO-Fe_3_O_4_-Ti^(IV)^ suspension was filtered, washed several times with dry CHCl_3_, and finally air-dried for 2 days.

### General procedure for synthesis of 2-aryl/alkyl-2,3-dihydro-1*H*-naphtho[1,2-*e*][1,3]oxazine

**Fig. 1:**
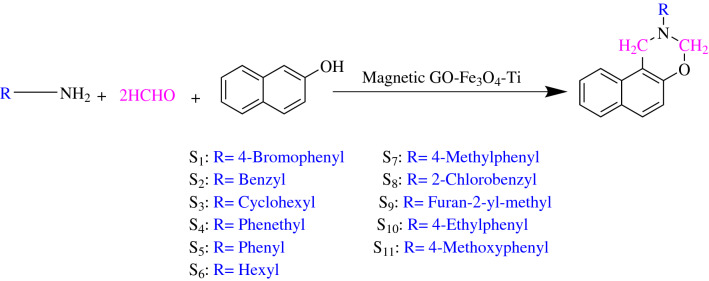
Synthesis of 2-aryl/alkyl-2,3-dihydro-1*H*-naphtho[1,2-*e*][1,3]oxazine derivatives ***(S***_***1***_***–S***_***11***_***)***

β-naphthol (1.0 mmol), primary amine (1.0 mmol), formaldehyde 37% (2.0 mmol/0.07 mL), and GO-Fe_3_O_4_-Ti ^(IV)^ (0.4 g) were added and heated at 65 °C without any solvent. After completing the reaction (monitored by TLC), the reaction mixture was dissolved in ethanol (3 mL) and the catalyst was separated by an external magnet. Afterward, cold water was added and the resulting precipitate was collected by filtration. Eventually, the obtained precipitate was recrystallized and purified from hot ethanol to get the final compounds ***(S***_***1***_***–S***_***11***_***)*** (Fig. 1). The recovered catalyst was washed three times with hot acetone, dried in the oven at 60 °C (one day), and reused for subsequent applications.

### Anticonvulsant evaluation via pentylenetetrazole (PTZ) seizure threshold test

Anticonvulsant effects of the synthesized naphto 1,3 oxazine derivatives were evaluated by using the PTZ model in male BALB/c strains of rats weighing from 25 to 30 g (obtained from the Pasteur Institute central animal house, Tehran, Iran). The animals were housed in polycarbonate cages under 25 ± 1 °C with a 12-h light/dark cycle. All animals were maintained in the animal house and had free access to water and food. The ethical guidelines for using experimental animals were followed in all tests under ethical committee acts of Iran University of Medical Sciences and the European Communities Council Directive 24 November 1986 (86/609/EEC). The animals were divided randomly into 3 groups (n = 4) as follows: (1) control group, received normal saline (10 mL/kg, i.p.) 30 min before administrating PTZ; (2) diazepam group, received diazepam (5 mg/kg, i.p.) 30 min before administrating PTZ; (3) received naphto 1,3 oxazines group (50 mg/kg and 100 mg/kg i.p.) 30 min before administrating PTZ. Afterward, seizure latency, seizure duration, and percentage of seizure protection in 30 min after injection of PTZ were recorded.

### Statistical analysis

Data are expressed as Mean ± SEM. All the values were analyzed using the one-way analysis of variance (ANOVA) followed by Dunnett's test. Statistical software was SPSS (IBM SPSS Statistics 26.0.0.1 FP001) and p < 0.05 was considered as a statistically significant value.

### Molecular docking study

3D X-ray crystal structures of GABA receptor (PDB ID: 6X3X) were regained from Protein Data Bank (http://www.rcsb.org). The chemical structure of all synthesized compounds was generated, minimized, and converted to pdbqt format. The inherent ligand, and water molecules were removed from the PDB format, and then, the missing hydrogen atoms were added, and eventually, non-polar hydrogens, according to their corresponding carbons, were merged. All preparation was performed by the AutoDock Tools package (1.5.6). The docking procedure was done at the grid box with a size of 30 × 30 × 30 and the center of x = 147.199, y = 120.984, z = 122.347 by AutoDock Vina (1.1.2) using an in-house batch script (DOCKFACE) [25, 26]. The exhaustiveness was set to 100, and other docking parameters were default. All interaction analysis and image preparations were performed by Discovery Studio Visualizer v16.1.0.152350.

### Molecular dynamic simulation

The molecular dynamics simulations of the best docking pose of lig10 and diazepam in complex with 6X3X were performed using the Gromacs simulation package version 5.1 [27]. Setting the atom types and generating of the force field parameters were prepared using the Amber99SB force field. Topology and coordinate files of the ***S***_***10***_ and diazepam were created using the ACPYPE webserver. The periodic cuboidal box of the size of 21 × 21 × 21 Å was filled with TIP3P water molecules and the sodium chloride counter ions were added to neutralize the systems, electrically. To minimize the energy of the systems the steepest descent algorithm for 50,000 steps with a cut-off value of 1000 kJ mol^−1^ was used. The whole system was equilibrated under the NVT and NPT ensemble through 100 ps time to control the temperature near 300 K. The pressure 1 bar via a V-rescale Berendsen thermostat Parrinello-Rahman barostat, respectively. Finally, the production MD of 120 ns was performed with a 2 fs time step on the well-equilibrated system at the temperature of 300 K and a pressure of 1 bar. The production MD of 120 ns was performed on the well-equilibrated system at the temperature of 300 K and a pressure of 1 bar. The periodic boundary simulation based on the particle mesh Ewald (PME) method was accomplished, and the covalent bond lengths were constrained by applying the SHAKE algorithm. Then, Root-Mean-Square Deviation (RMSD), residue Root Mean Square Fluctuation (RMSF), radio use of gyration, and the number of hydrogen bonds between ligands and protein were analyzed to monitor the stability of the ligand–protein complex during the simulation and to define the equilibrium time of the simulation. Molecular mechanics Poisson–Boltzmann surface area (MM-PBSA) method has been used to evaluate ligand-binding affinities in the systems. MD simulation results containing trajectories were studied using the Visual Molecular Dynamics (VMD) version 1.8.7 and discovery studio software v16.1.0.152350 were employed for simulation interaction between target molecules and protein [28, 29].

## Result and discussion

Design of effective and economical chemical procedures using heterogeneous catalysts to prepare pharmaceutical products has recently gained noteworthy interest [19]. Magnetite nanoparticles also have attracted remarkable attention as a powerful catalyst support due to their unique characteristics [20]. Heterogeneous catalysts containing titanium has a strong tendency to react with oxygen. So it can be used as a strong Lewis acid catalyst to react with oxygen containing compounds. In this regards, firstly, GO was achieved according to the Hummers method. Fe_3_O_4_, through the chemical co-precipitation of Fe^2+^ and Fe^3+^ ions in a basic solution, was then exposed to GO. Eventually, the TiCl_4_ was used to surface modification of GO-Fe_3_O_4_ to obtain magnetic composite (GO-Fe_3_O_4_-Ti^(IV)^), which is appropriate as a catalyst in MCR reactions and also, illustrated high stability and great catalytic performance in the synthesis of naphto 1,3 oxazine derivatives ***(S***_***1***_***–S***_***11***_***)***. The reaction conditions were also optimized based on temperature, solvent, and amounts of catalyst. After exerting the catalyst in the chemical reactions, it could be separated and reused 5 times without any significant decrease in its catalytic capacity. The chemical structures of ***S***_***1***_***–S***_***11***_ were confirmed by IR, ^1^H-NMR and ^13^C-NMR. In IR analysis, the stretching frequency of the C=C bond was observed at 1623–1467 cm^−1^ and also, the stretching frequencies of C–O and C–N were seen at 1096–1031 and 1231–1223, respectively. The significant piece of the ^1^H NMR spectrum of compounds ***S***_***1***_***-S***_***11***_ was a single peak at 4.87–5.54 and 4.25–5.06 ppm related to the two protons at positions 2 and 4 of the oxazine ring.

### Catalyst identification

#### Fourier transform infrared (FT-IR) analysis

**Fig. 2:**
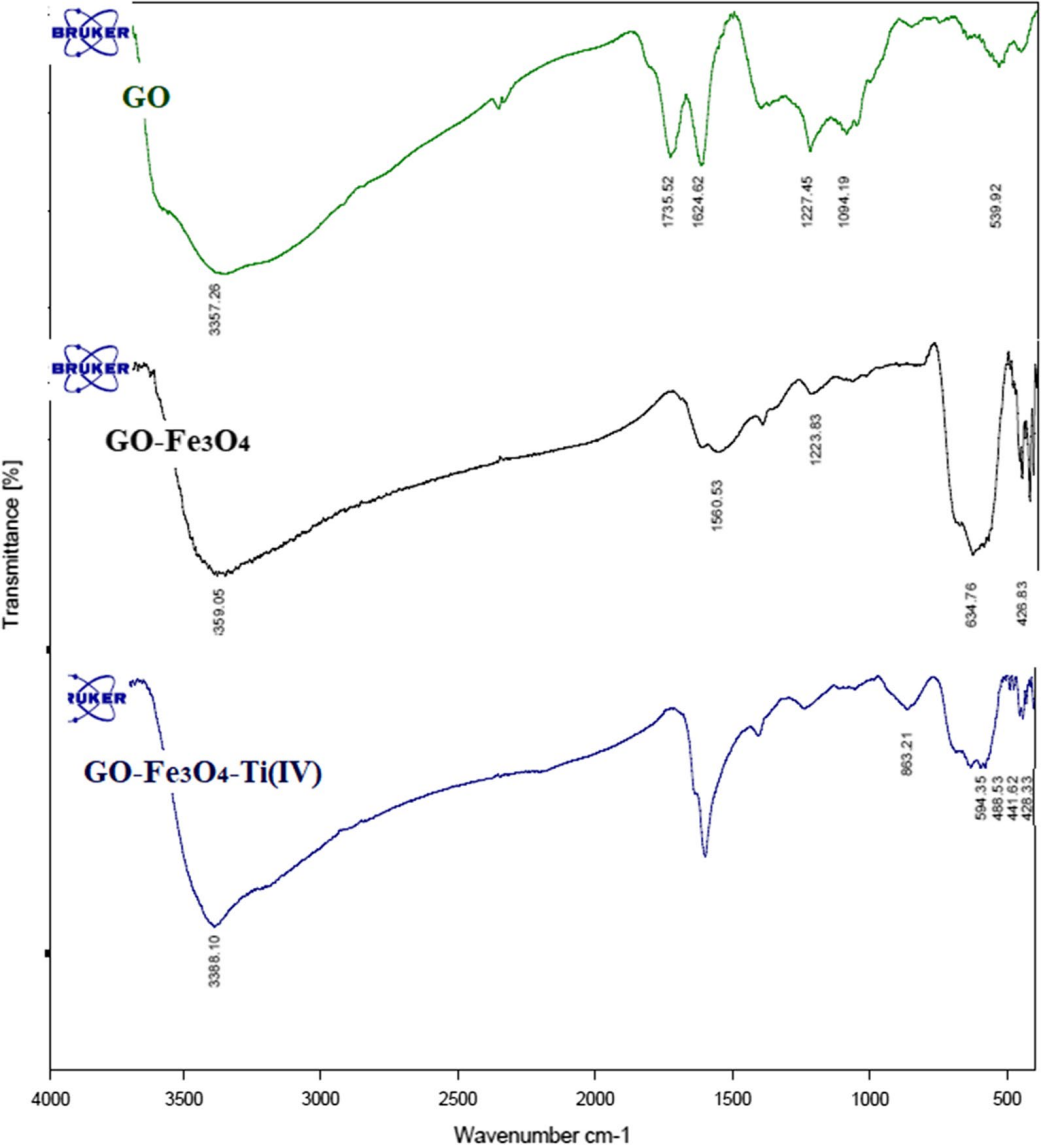
FT-IR spectrum of GO, GO-Fe_3_O_4_, and GO-Fe_3_O_4_-Ti^(IV)^

The FT-IR spectrums of GO, GO-Fe_3_O_4_, and GO-Fe_3_O_4_–Ti^(IV)^ were presented in Fig. 2. The primary FT-IR spectrum of GO illustrated absorption peaks in 3357, 1736, and 1625 cm^−1^, which are related to the stretching bond of OH groups, the stretching bonds of C=O of the carboxylic acid, and the stretching vibration of C=C, respectively. The peaks observed in GO confirm the existence of functional groups in this compound. The sharp peaks at about 625 cm^−1^ of GO-Fe_3_O_4_ and GO-Fe_3_O_4_-Ti^(IV)^ are related to the bending bond of Fe–O which confirms the presence of Fe_3_O_4_ in the structure of this composite. The peak at 1625 cm^−1^ is attributed to the overlapping of the deformation of the O–H bond in the strongly intercalated water absorbed by GO and the aromatic skeletal C=C stretching vibration of the GO sheets. No specific IR signal is viewed to illustrate the presence of Ti^(IV)^ on the GO-Fe_3_O_4_ nanocomposites spectrum. However, the decrease in peak intensity observed in 625 cm^−1^ of GO-Fe_3_O_4_-Ti^(IV)^ is probably due to its titanium covering. To confirm the presence of titanium in the composite structure, the EDS analysis was done and its results showed the existence of C, N, O, Fe, and Ti elements in the prepared catalyst. It was also distinguished that Ti had been well located on the surface of GO (Additional file 1: Fig. S33). The VSM analysis of the GO-Fe_3_O_4_-Ti^(IV)^ magnetic nanocomposite was shown in Additional file 1: Fig. S33. The saturation magnetization of the magnetic nanocomposite is smaller compared to the pure Fe_3_O_4_ which due to binding of Ti^(IV)^ on the Fe_3_O_4_ particles. However, GO-Fe_3_O_4_-Ti^(IV)^ could be disported with an external magnet from the reaction mix (Additional file 1: Fig. S34).

#### XRD analysis

**Fig. 3:**
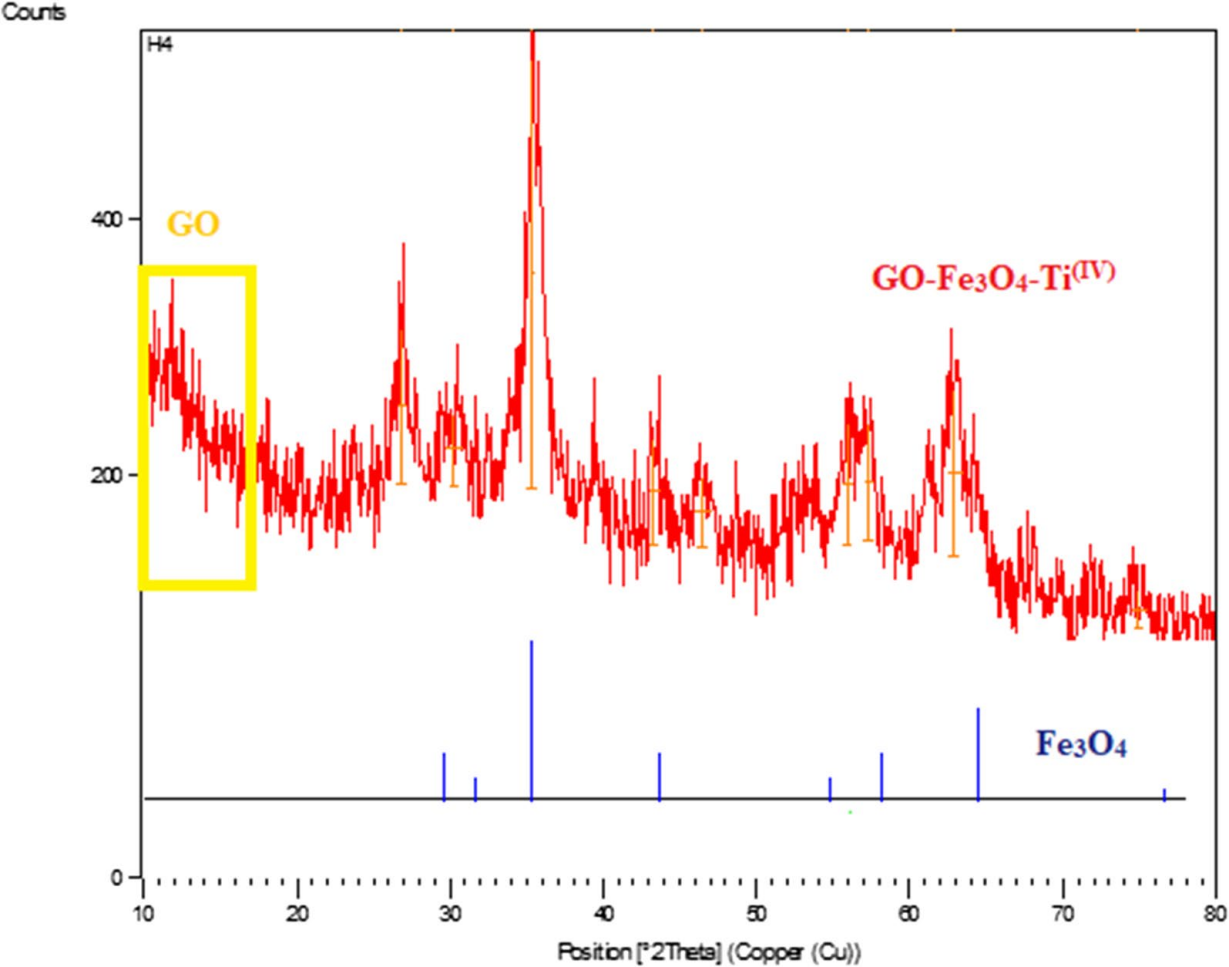
XRD patterns of GO-Fe_3_O_4_-Ti^(IV)^

The XRD pattern of GO-Fe_3_O_4_-Ti^(IV)^ was shown in Fig. 3. In this Figure, the wide peak at 2θ = 10–15° which is indicated by yellow box is related to the graphene oxide. The diffraction peaks at 2θ = 29.9, 35.6, 43.2, 57.2, and 62.7° are in accordance with the pure spinel of Fe_3_O_4_. Also, the peak at 2θ = 28.0° represented the bond between titanium and oxygen in the composite sheet. The presence of these diffractions in the XRD pattern indicated that the crystal structure of Fe_3_O_4_ is well preserved [23].

#### Optimization of the reaction conditions

The efficacy performance of GO-Fe_3_O_4_-Ti^(IV)^ as a catalyst for the synthesis of naphto 1, 3 oxazine derivatives were done through the reaction of 4-methoxy aniline (1 mmol), formaldehyde (2 mmol) and 2-naphtol (1 mmol) to get ***S***_***11***_ as a model reaction. To optimize the reaction conditions, different parameters such as type and amounts of catalyst, solvent and temperature were tested to obtain the most favorable conditions (Table 1). To achieve the efficacy of the catalyst, the reaction was implemented with GO, GO-Fe_3_O_4_, GO-Fe_3_O_4_-Ti^(IV)^ and GO-Ti^(IV)^ under solvent-free conditions through entries 1–4. The best result was observed through the presence of Ti^(IV)^ on the surface of GO-Fe_3_O_4_. To find out the best solvent, different media such as ethanol, water, chloroform as well as solvent-free were examined. The best suitable media was generated under solvent-free with 92% efficiency (Table 1, entries 5–8). In the next step, to optimize the temperature, the reaction was performed in the range of room temperature to 100 °C (Table 1, entries 8–10). 60 °C was determined as the best temperature. Finally, the reaction was performed in different amounts of GO-Fe_3_O_4_-Ti^(IV)^ (Table 1, entries 11–14). The best result was observed with 0.04 g of catalyst. Eventually, a catalyst value of 0.04 g, no solvent at 60 °C, was selected as the best condition for synthesizing the other derivatives.

**Table 1:** Optimization of the reaction conditions for the synthesis of ***S***_***11***_


Entry	Catalyst (g)	Temp	Solvent	Time (min)	Yield (%)
1	GO (0.04)	60	Solvent-free	35	30
2	GO-Fe_3_O_4_ (0.04)	60	Solvent-free	30	65
3	GO-Fe_3_O_4_-Ti ^(IV)^ (0.04)	60	Solvent-free	12	92
4	GO-Ti ^(IV)^ (0.04)	60	Solvent-free	40	85
5	GO-Fe_3_O_4_-Ti ^(IV)^ (0.04)	60	C_2_H_5_OH	23	85
6	GO-Fe_3_O_4_-Ti ^(IV)^ (0.04)	60	H_2_O	25	85
7	GO-Fe_3_O_4_-Ti ^(IV)^ (0.04)	60	CHCl_3_	25	48
8	GO-Fe_3_O_4_-Ti ^(IV)^ (0.04)	60	Solvent-free	12	92
9	GO-Fe_3_O_4_-Ti ^(IV)^ (0.04)	Room temperature	Solvent-free	50	70
10	GO-Fe_3_O_4_-Ti ^(IV)^ (0.04)	100	Solvent-free	12	92
11	GO-Fe_3_O_4_-Ti ^(IV)^ (0.01)	60	Solvent-free	40	67
12	GO-Fe_3_O_4_-Ti ^(IV)^ (0.02)	60	Solvent-free	45	85
13	GO-Fe_3_O_4_-Ti ^(IV)^ (0.03)	60	Solvent-free	20	90
14	GO-Fe_3_O_4_-Ti ^(IV)^ (0.05)	60	Solvent-free	12	92

#### Catalytic activity of the catalyst

**Fig. 4:**
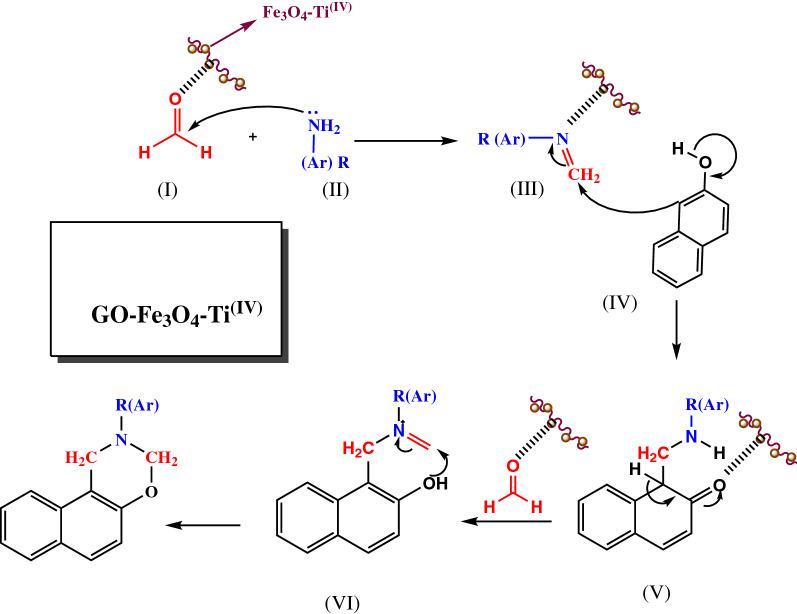
Proposed mechanism for synthesis of 2-(aryl or alkyl)-2,3-dihydro-1H-naphtho[1,2-e][1,3]oxazine

After optimization of the reaction conditions, the effectiveness of GO-Fe_3_O_4_-Ti ^(IV)^ was proved by a one-pot synthesis reaction among various naphto-1,3-oxazine derivatives. The results showed this catalytic system was successfully converted the various primary amines (aliphatic and aromatic) to desired products with high-efficiency yields (Table 2). The proposed mechanistic pathways for the synthesis of 2-(aryl or alkyl)-2,3-dihydro-1H-naphtho[1,2-e][1,3]oxazine derivatives was shown in Fig. 4.

**Table 2:** Chemical structures, spectra data, and yields of the final synthesized of 1,3-naphtoxazines

Entry	Chemical name	Spectra data	Yield (%)
***S***_***1***_	2-(4-bromophenyl)-2,3-dihydro-1H-naphtho[1,2-e][1,3]oxazine	White solid, m.p. 116–118 °C. FT-IR (ATR) ῡ (cm^−1^): 1588, 1489, 1223, 1092. ^1^H NMR (Acetone-d_6_, 400 MHz) δ ppm: 7.84 (t, 2H, J = 9.2 Hz, Ar–H), 7.71 (d, 1H, J = 9.2 Hz, Ar–H), 7.52 (t, 1H, J = 8.4 Hz, Ar–H), 7.37–7.40 (m, 3H, Ar–H), 7.20 (d, 2H, J = 9.2 Hz, Ar–H), 7.01 (d, 1H, J = 9.2 Hz, Ar–H), 5.51 (s, 2H, O–CH_2_–N), 5.03 (s, 2H, –Ar–CH_2_–N)	95
***S***_***2***_	2-benzyl-2,3-dihydro-1H-naphtho[1,2-e][1,3]oxazine	Off-white, m.p. 125–127 °C. FT-IR (ATR) ῡ (cm^−1^): 1623, 1597, 1461, 1223, 1058, 738. ^1^H NMR (DMSO-d_6_, 500 MHz) δ ppm: 7.05–7.71 (m, 11H, Ar–H), 4.89 (s, 2H, O–CH_2_–N), 4.22 (s, 2H, –Ar–CH_2_–N), 3.89 (s, 2H, –Ar–CH_2_–N). ^13^C NMR (DMSO-d_6_, 100 MHz) δ ppm: 46.5, 55.2, 81.5, 111.7, 118.3, 121.1, 123.4, 126.6, 127.2, 127.8, 128.3, 128.4, 128.5, 128.6, 131.5, 138.3, 151.4	94
***S***_***3***_	2-cyclohexyl-2,3-dihydro-1H-naphtho[1,2-e][1,3]oxazine	Off-white solid, m.p. 248 °C (d). FT-IR (ATR) ῡ (cm^−1^): 2926, 2852, 1597, 1467, 1227, 1058. ^1^H NMR (DMSO-d_6_, 500 MHz) δ ppm: 7.66–7.81 (m, 3H, Ar–H), 7.48 (m, 1H, Ar–H), 7.35 (m, 1H, Ar–H), 6.98 (m, 1H, Ar–H), 4.99 (s, 2H, O–CH_2_–N), 4.33 (s, 2H, –Ar–CH_2_–N), 2.70 (m, 1H, CH–N), 1.08–1.86 (m, 10H, 5CH_2_)	92
***S***_***4***_	2-phenethyl-2,3-dihydro-1H-naphtho[1,2-e][1,3]oxazine	White solid, m.p. 232 °C (d). FT-IR (ATR) ῡ (cm^−1^): 1597, 1469, 1225, 1060. ^1^H NMR (DMSO-d_6_, 500 MHz) δ ppm: 7.81 (m, 1H, Ar–H), 7.69 (m, 2H, Ar–H), 7.49 (m, 1H, Ar–H), 7.35 (m, 1H, Ar–H), 7.24 (m, 5H, Ar–H), 7.02 (m, 1H, Ar–H), 4.92 (s, 2H, O-CH_2_-N), 4.32 (s, 2H, –Ar–CH_2_–N), 2.96 (m, 2H, Ar–CH_2_–CH_2_–N), 2.88 (m, 2H, Ar–CH_2_–CH_2_–N). ^13^C NMR (DMSO-d_6_, 125 MHz) δ ppm: 34.9, 47.9, 54.0, 82.7, 113.1, 119.2, 122.3, 124.2, 126.7, 127.3, 128.5, 129.1, 129.2, 129.3, 129.5, 132.4, 140.9, 152.4	92
***S***_***5***_	2-phenyl-2,3-dihydro-1H-naphtho[1,2-e][1,3]oxazine	White solid, m.p. 47–49 °C FT-IR (ATR) ῡ (cm^−1^): 3060, 1600, 1496, 1231, 1057; ^1^H NMR (Acetone-d_6_, 400 MHz) δ ppm: 7.89 (d, 1H, J = 8.4 Hz, Ar–H), 7.85 (d, 1H, J = 8 Hz, Ar–H), 7.73 (d, 1H, J = 9.2 Hz, Ar–H), 7.53–7.57 (m, 1H, Ar–H), 7.38–7.42 (m, 1H, Ar–H), 7.23–7.28 (m, 4H, Ar–H), 7.04 (d, 1H, J = 8.8 Hz, Ar–H), 6.87–6.91 (m, 1H, Ar–H), 5.54 (s, 2H, O–CH_2_–N), 5.06 (s, 2H, –Ar–CH_2_–N)	91
***S***_***6***_	2-hexyl-2,3,4a,10b-tetrahydro-1H-naphtho[1,2-e][1,3]oxazine	Brown solid, m.p. 177 °C (d). FT-IR (ATR) ῡ (cm^−1^): 2927, 2854, 1597, 1467, 1225, 1057. ^1^H NMR (DMSO-d_6_, 500 MHz) δ ppm: 7.80 (m, 1H, Ar–H), 7.68 (m, 2H, Ar–H), 7.47 (m, 1H, Ar–H), 7.34 (m, 1H, Ar–H), 7.00 (m, 1H, Ar–H), 4.87 (s, 2H, O–CH_2_–N), 4.25 (s, 2H, –Ar–CH_2_–N), 2.68 (m, 2H, –CH_2_–N), 1.53 (m, 2H, CH_2_), 1.25 (m, 6H, 3CH_2_), 0.84 (m, 3H, CH_3_). ^13^C NMR (DMSO-d_6_, 125 MHz) δ ppm: 14.8, 23.0, 27.2, 28.4, 32.0, 47.9, 52.1, 82.7, 113.0, 119.1, 122.2, 124.1, 127.3, 128.5, 129.2, 129.3, 132.4, 152.4	91
***S***_***7***_	2-(p-tolyl)-2,3-dihydro-1H-naphtho[1,2-e][1,3]oxazine	Yellow solid, m.p. 87–89 °C. FT-IR (ATR) ῡ (cm^−1^): 1597, 1470, 1226, 1096. ^1^H NMR (Acetone-d_6_, 400 MHz) δ ppm: 7.82 (t, 2H, J = 8.8 Hz, Ar–H), 7.69 (d, 1H, J = 9.2 Hz, Ar–H), 7.51 (t, 1H, J = 8 Hz, Ar–H), 7.36 (t, 1H, J = 8 Hz, Ar–H), 6.99–7.04 (m, 3H, Ar–H), 7.09–7.11 (m, 2H, Ar–H), 5.52 (s, 2H, O–CH_2_–N), 5.02 (s, 2H, –Ar–CH_2_–N), 2.24 (s, 3H, CH_3_–Ar)	94
***S***_***8***_	2-(2-chlorobenzyl)-2,3-dihydro-1H-naphtho[1,2-e][1,3]oxazine	Yellow soid, m.p. 70–75 °C. FT-IR (ATR) ῡ (cm^−1^): 1594, 1468, 1226, 1057. ^1^H NMR (DMSO-d_6_, 500 MHz) δ ppm: 7.07–7.82 (m, 10H, Ar–H), 4.95 (s, 2H, O–CH_2_–N), 4.27 (s, 2H, –Ar–CH_2_–N), 4.00 (s, 2H, –Ar–CH_2_–N). ^13^C NMR (DMSO-d_6_, 100 MHz) δ ppm: 46.8, 52.6, 82.0, 111.7, 118.3, 121.3, 123.4, 126.5, 127.2, 127.8, 128.4, 128.5, 128.9, 129.35, 130.6, 131.5, 133.2, 135.8, 151.4	85
***S***_***9***_	2-(furan-2-ylmethyl)-2,3-dihydro-1H-naphtho[1,2-e][1,3]oxazine	Pale-pink, m.p. 98–100 °C. FT-IR (ATR) ῡ (cm^−1^): 1597, 1467, 1226, 1060. ^1^H NMR (DMSO-d_6_, 500 MHz) δ ppm: 7.71–8.02 (m, 4H, Ar–H), 7.36–7.61 (m, 2H, Ar–H), 7.04 (s, 1H, Ar–H), 6.32–6.52 (m, 2H, Ar–H), 4.89 (s, 2H, O–CH_2_–N), 4.26 (s, 2H, –Ar–CH_2_–N), 3.90 (s, 2H, furan-CH_2_–N). ^13^C NMR (DMSO-d_6_, 125 MHz) δ ppm: 46.4, 47.9, 81.2, 108.8, 110.4, 111.5, 118.3, 121.2, 123.4, 126.6, 127.8, 128.4, 128.5, 131.48, 142.7, 151.3, 151.8	90
***S***_***10***_	2-(4-ethylphenyl)-2,3-dihydro-1H-naphtho[1,2-e][1,3]oxazine	Off-white solid, m.p. 43–46 °C. FT-IR (ATR) ῡ (cm^−1^): 1597, 1467, 1226, 1056. ^1^H NMR (Acetone-d_6_, 400 MHz) δ ppm: 7.80–7.85 (m, 2H, Ar–H), 7.69–7.71 (m, 1H, Ar–H), 7.50–7.55 (m, 1H, Ar–H), 7.35–7.40 (m, 1H, Ar–H), 6.99–7.14 (m, 5H, Ar–H), 5.49 (s, 2H, O-CH_2_-N), 5.00 (s, 2H, -Ar-CH_2_-N), 2.49–2.54 (m, 2H, –CH_2_–CH_3_), 1.15 (t, 3H, J = 7 Hz, –CH_2_–CH_3_)	89
***S***_***11***_	2-(4-methoxyphenyl)-2,3-dihydro-1H-naphtho[1,2-e][1,3]oxazine	Off-white solid, m.p. 76–78 °C FT-IR (ATR) ῡ (cm^−1^): 1596, 1467, 1246, 1230, 1031. ^1^H NMR (Acetone-d_6_, 400 MHz) /δ ppm: 7.80–7.82 (m, 2H, Ar–H), 7.70 (d, 1H, J = 8.8 Hz, Ar–H), 7.51 (m, 1H, Ar–H), 7.36 (m, 1H, Ar–H), 7.13 (d, 2H, J = 7.2 Hz, Ar–H), 7.00 (d, 1H, J = 8.8 Hz, Ar–H), 6.80 (d, 2H, J = 7.2 Hz, Ar–H), 5.42 (s, 2H, O-CH_2_-N), 4.93 (s, 2H, -Ar-CH_2_-N), 3.68 (s, 3H, O-CH_3_)	92

The intermediate III was achieved from the Mannich condensation of the different substituted amines (II) and formaldehyde (I). Secondly, a nucleophilic attack occurred between 2-naphthol and the intermediate III. Finally, the target compounds were achieved with an intramolecular cyclization of intermediate (VI) (Fig. 3).

The catalytic role of GO-Fe_3_O_4_-Ti^(IV)^ in 2-(aryl or alkyl)-2,3-dihydro-1H-naphtho[1,2-e][1, 3] oxazine synthesis was compared with other reported catalysts to evaluate the potency of our catalyst. The results illustrated the most capability of GO-Fe_3_O_4_-Ti^(IV)^ as a catalyst (Table 3).

**Table 3:** Comparison between different methods for synthesis of 2-(aryl or alkyl)-2,3-dihydro-1H-naphtho[1,2-e][1,3]oxazine derivatives

Entry	Conditions	Yield (%)	Refs.
	Catalyst/Temp/Time/Solvent		
1	Nano-Al_2_O_3_/BF_3_/Fe_3_O_4_/R.T/20 min/Water	92	[6]
2	Fe_3_O_4_@nano-dextrin/Ti^(IV)/^R.T/7 min/Water	95	[33]
	Fe_3_O_4_@NCs/TiCl/R.T/3 min/solvent-free	98	[35]
3	Nano-Fe_3_O_4_@walnut shell/Cu(II)/60 °C/25 min/solvent-free	93	[34]
4	GO-Fe_3_O_4_-Ti^(IV)/^60 °C/15 min/solvent-free	97	This work

According to Table 3, calm reaction conditions, proper yields, short reaction time, easy and more convenient a catalyst separation and workup are some of the advantages of this method compared to the other previous works [16, 30–34].

#### Catalyst recovery and reuse

**Fig. 5:**
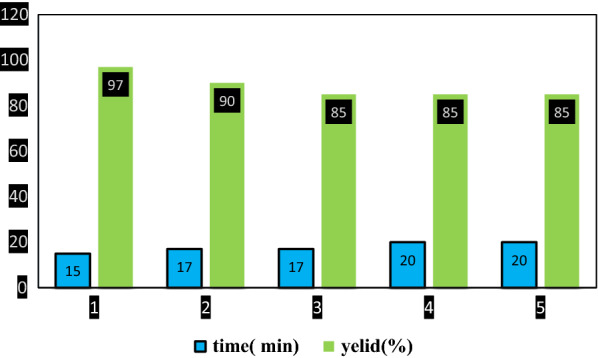
Recyclability of the GO-Fe_3_O_4_-Ti^(IV)^ in the synthesis of ***S***_***11***_

The synthesis of ***S***_***11***_ as a model reaction was done to investigate the recyclability of the GO-Fe_3_O_4_-Ti^(IV)^. After completing the reaction, the catalyst was separated from the reaction mixture using an external magnet and washed with hot acetone. Finally, it dried at room temperature and then used for the next run. The recycled catalyst was reused in the mentioned reaction four times without a considerable decrease in its catalytic activity (Fig. 5). A low difference in the efficiency may be due to the detached of titanium from the surface of the catalyst or the contamination of the catalyst surface.

**Fig. 6:**
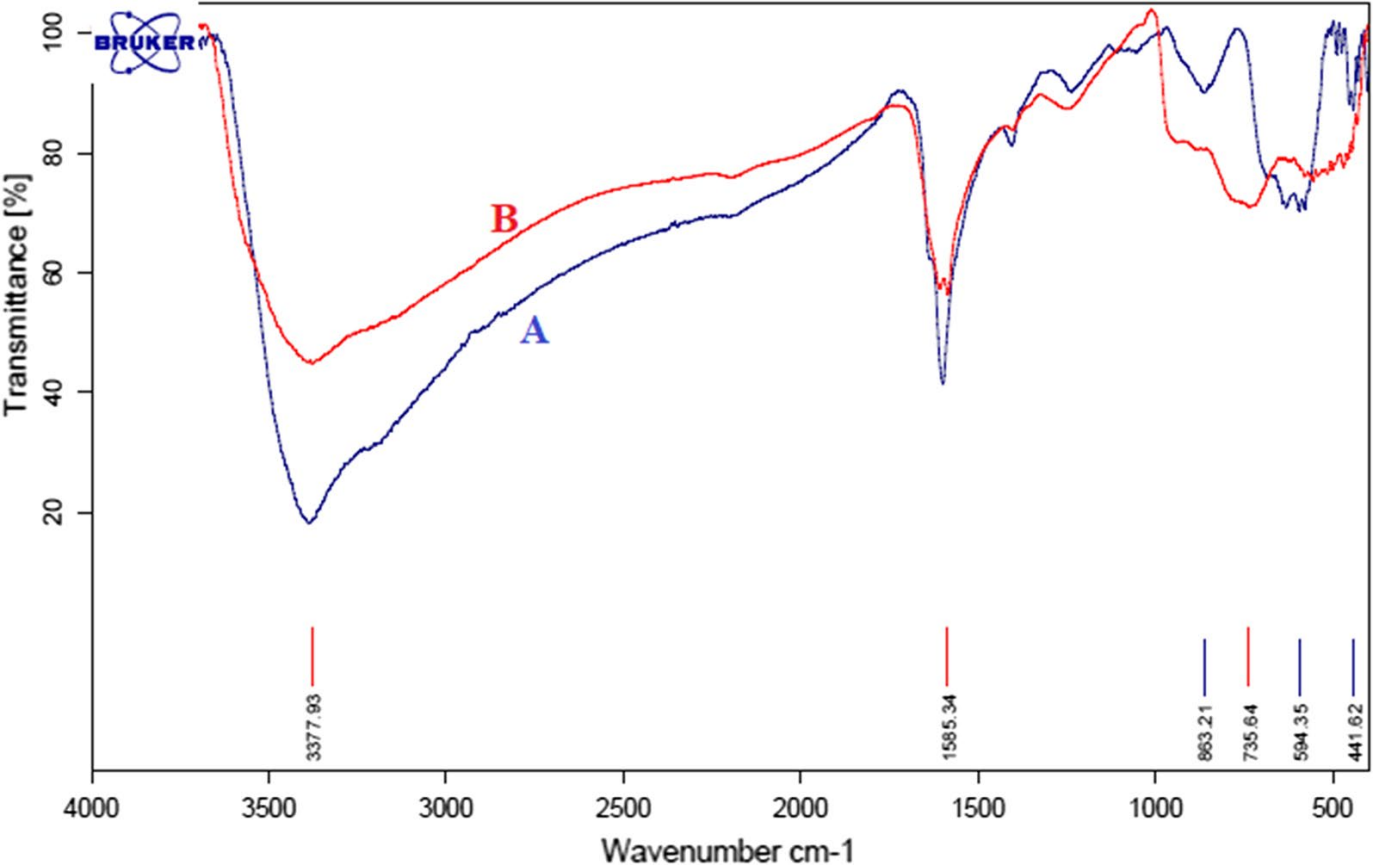
Comparison of the FT-IR spectrum of the GO-Fe_3_O_4_-Ti^(IV)^ before (*A*) and after the recycling (*B*)

Figure 6 represents the difference between the FT-IR spectra before and after the recycling. No significant changes were seen. The peak observed at 735 regions may be due to impurity created on the catalyst, which has little effect on the strength of the catalyst in the reaction.

### Anticonvulsant activity

**Fig. 7:**
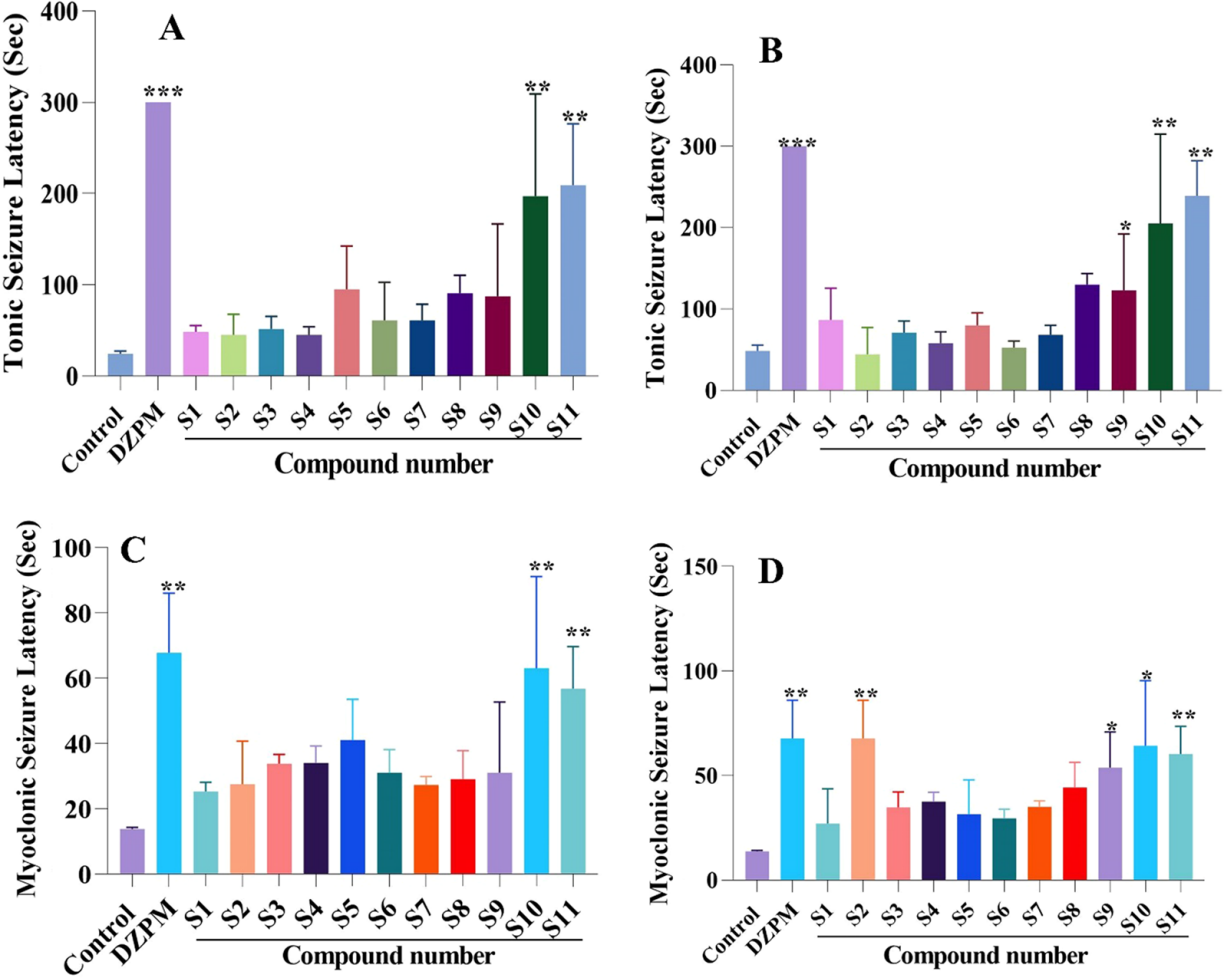
Effect of 2-(aryl or alkyl)-2,3-dihydro-1H-naphtho[1,2-e][1,3]oxazine derivatives on tonic at dose of 50 mg/kg (**A**) and 100 mg/kg (**B**) and myoclonic at dose of 50 mg/kg (**C**) and 100 mg/kg (D) seizure latency. Data are mean ± standard error of the mean of the latency time, (n = 6–8). *P < 0.05, **P < 0.01 and ***P < 0.001

All the synthesized 2-(aryl or alkyl)-2,3-dihydro-1H-naphtho[1,2-e][1, 3] oxazine derivatives were screened for the anticonvulsant properties to recognize, the compounds that increase the seizure thresholds in PTZ induced seizure model. Two doses of all compounds (50 and 100 mg/kg) were administrated and their effects on intraperitoneal PTZ-induced myoclonic and tonic seizure threshold are shown in Fig. 7. Diazepam (5 mg/kg) was used as a positive control. At tonic seizure, compound ***S***_***11***_ which possessed an electron donating group (methoxy) on the phenyl group of the amine moiety, increased the seizure threshold in both doses significantly and is considered as the most potent compound. This higher activity might be due to the more favorable orientation of the compound inside the active site of the GABA receptor. Also, compounds ***S***_***10***_ and ***S***_***9***_ in 50 and 100 mg/kg elevated the seizure threshold compared to the vehicle group. At myoclonic seizure, compounds ***S***_***10***_ and ***S***_***11***_ increased the seizure threshold in the i.p. PTZ model at a 50 mg/kg dose. However, compounds ***S***_***2***_*, ****S***_***9***_*, ****S***_***10***_ and ***S***_***11***_ demonstrated significant anticonvulsant effects in doses of 100 mg/kg. Therefore, it could be suggested that the presence of electron donating groups such as methoxy *(****S***_***11***_*)* and ethyl *(****S***_***10***_*)* in cases of compounds in which the phenyl group directly placed on the oxazine ring could increase the anticonvulsant effect. However, in the case of compounds containing methyl linker such as ***S***_***2***_ with no substituent on phenyl ring represented the best effect at dose of 100 mg/kg, while locating the chlorine atom of phenyl ring, in compound ***S***_***8***_***,*** led to diminish the activity. On the other hand, derivatives with ethyl linker such as compound ***S***_***4***_ and compound with aliphatic group *(****S***_***6***_*)* could decreased the anticonvulsant activity.

### In silico physicochemical parameters (ADME) prediction

The drug-likeness and pharmacokinetic properties of all synthesized compounds were represented in Tables 4 and 5 by Swiss ADME and PreADMET software. All compounds have a molecular weight in the range of 261–340. The other properties such as the number of rotatable bonds (n-RB), HBD, and HBA as hydrogen bond properties were within the acceptable limit which represented that all synthesized compounds had desired conformational flexibility. On the other hand, all compounds have TPSA in the region of 12.47–32.67 Å. which desires for acceptable penetration to the blood–brain barrier (BBB). They had desired lipophilicity (Log P) properties to diffuse into the cell membrane and so, they fulfilled the rule of five Lipinski’s rule.

**Table 4:** Physicochemical properties of the 2-(aryl or alkyl)-2,3-dihydro-1H-naphtho[1,2-e][1,3]oxazine derivatives

Compounds	MW^a^	LogP^b^	HBD^c^	HBA^d^	TPSA (A^2^) ^e^	n-RB^f^	Lipinski violation
***S***_***1***_	340.21	4.37	0	1	12.47	1	1
***S***_***2***_	275.34	3.72	0	2	12.47	2	0
***S***_***3***_	267.37	3.72	0	2	12.47	1	0
***S***_***4***_	289.37	3.95	0	2	12.47	3	0
***S***_***5***_	261.32	3.75	0	1	12.47	1	0
***S***_***6***_	269.38	3.72	0	2	12.47	5	0
***S***_***7***_	275.34	3.99	0	1	12.47	1	1
***S***_***8***_	309.79	4.21	0	2	12.47	2	1
***S***_***9***_	265.31	2.41	0	3	25.61	2	0
***S***_***10***_	289.37	4.21	0	1	12.47	2	1
***S***_***11***_	291.34	3.37	0	2	21.7	2	0
***Diazepam***	284.74	2.67	0	2	32.67	1	0
Rule of Lipinski	≤ 500	≤ 5	≤ 5	≤ 10	≤ 140	≤ 10	≤ 1

^a^ Molecular weight (MW); ^b^ Logarithm of partition coefficient between n-octanol and water (LogP); ^c^ Number of hydrogen bond donors (HBD); ^d^ Number of hydrogen bond acceptors (HBA); ^e^ Topological polar surface area (TPSA); ^f^ Number of rotatable bonds (nRB)

**Table 5:** In silico ADME of the 2-(aryl or alkyl)-2,3-dihydro-1H-naphtho[1,2-e][1,3]oxazine derivatives

Entry	Absorption			Distribution	
	% HIA^a^	In vitro Caco-2 cell permeability (nm s^−1^)	In vitro Skin permeability((log Kp, cm h − 1)	% In vitro plasma protein bonding	%BBB^b^
***S***_***1***_	100	57.30	− 2.68	100	2.27
***S***_***2***_	100	58.05	− 2.7	83.88	2.02
***S***_***3***_	100	58.16	− 3.07	83.09	4.07
***S***_***4***_	100	58.02	− 2.56	86.52	1.67
***S***_***5***_	100	58.15	− 2.74	91.96	2.07
***S***_***6***_	100	57.98	− 2.49	87.68	4.67
***S***_***7***_	100	58.15	− 2.66	92.29	3.22
***S***_***8***_	100	57.49	− 2.75	86.65	1.87
***S***_***9***_	100	56.37	− 3.33	82.78	2.86
***S***_***10***_	100	58.04	− 2.49	94.16	4.41
***S***_***11***_	100	57.65	− 2.96	92.59	0.37
***Diazepam***	99.49	47.68	− 3.07	98.74	2.58

^a^Human Intestinal Absorption; ^b^In vivo blood–brain barrier penetration

As it was mentioned in Table 5, all of the compounds are predicted to show well intestinal absorption (HIA) with the desired binding ability to plasma proteins (PPB) (more than 83). Regarding Caco-2 cell permeability, all compounds illustrated appropriate values in comparison to diazepam for penetration to biological membranes. Also, all of the compounds have a BBB value of more than 0.1 which showed these derivatives, could consider as CNS agents for epilepsy therapy.

### Molecular docking study

**Fig. 8:**
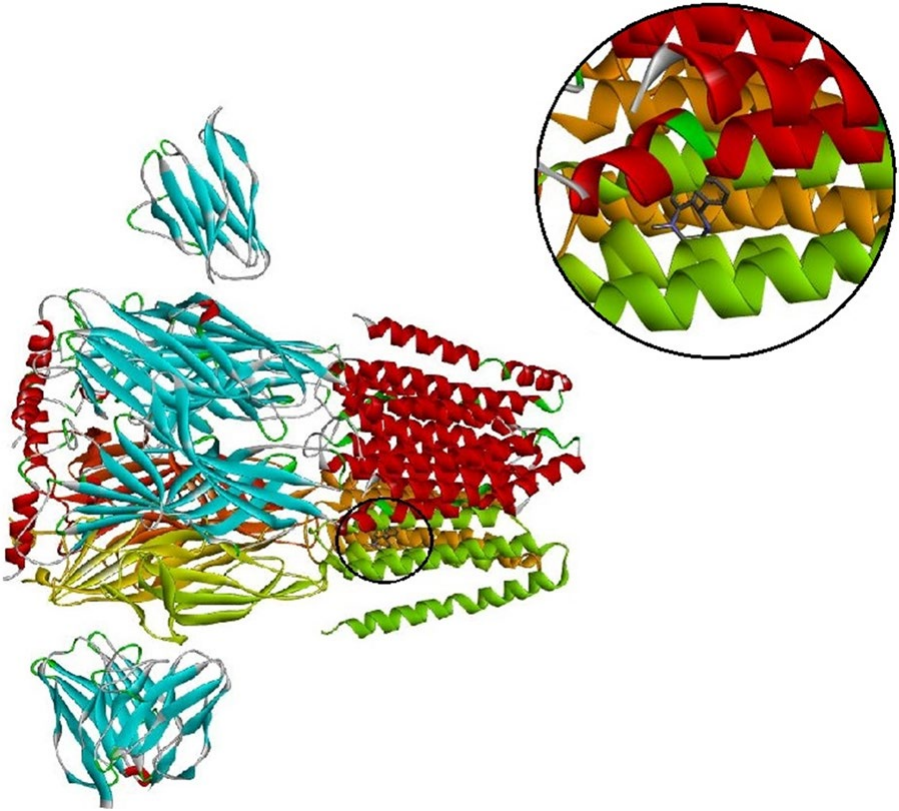
The full structure and binding pocket of GABA-A receptor (6X3X)

**Fig. 9:**
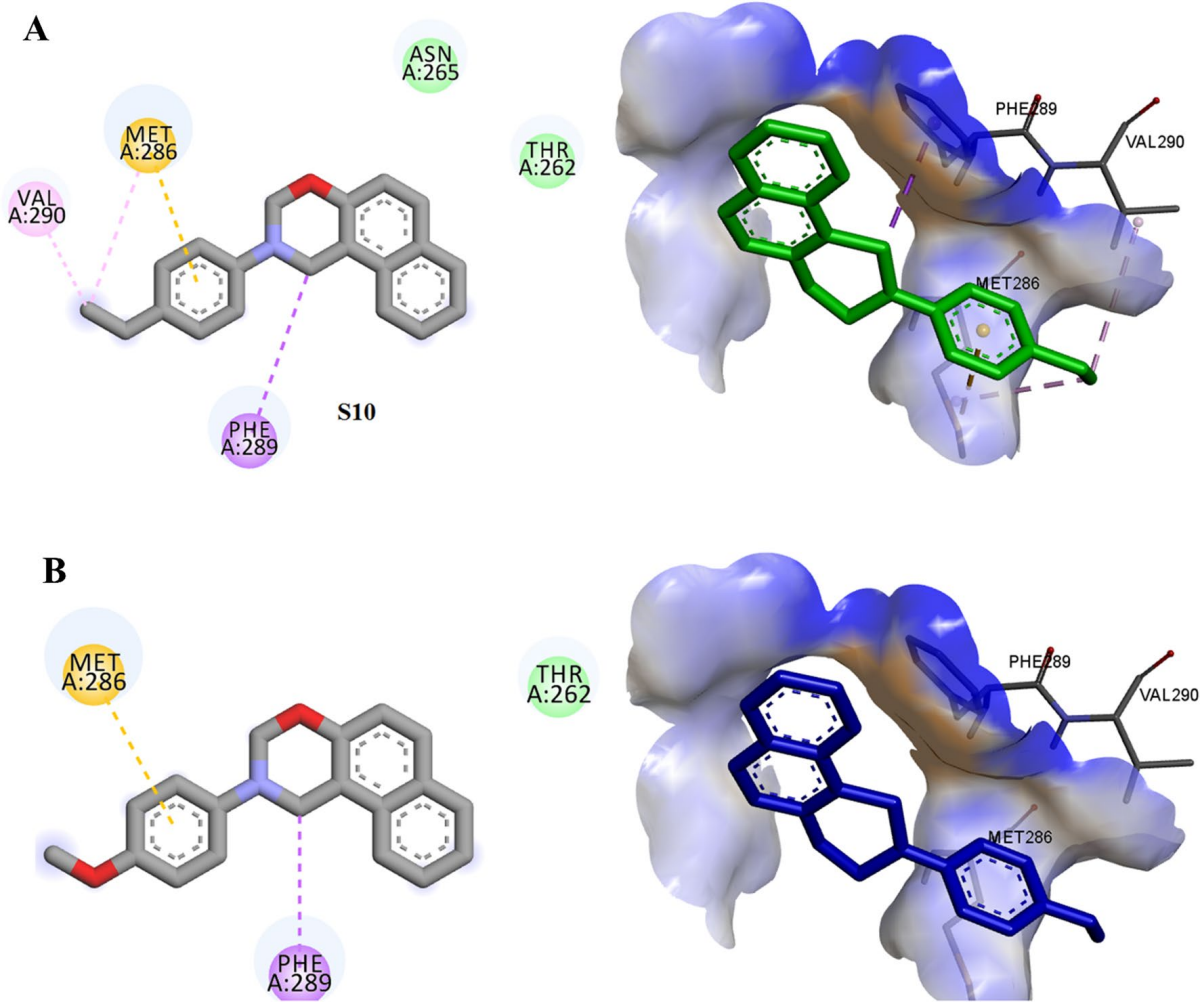
Interactions of ***S***_***10***_ (**A**) and ***S***_***11***_ (**B**) with the residues in the binding site of GABA-A receptor (orange: pi-sulfur, purple: pi-alkyl, green: van der Waals interaction)

**Fig. 10:**
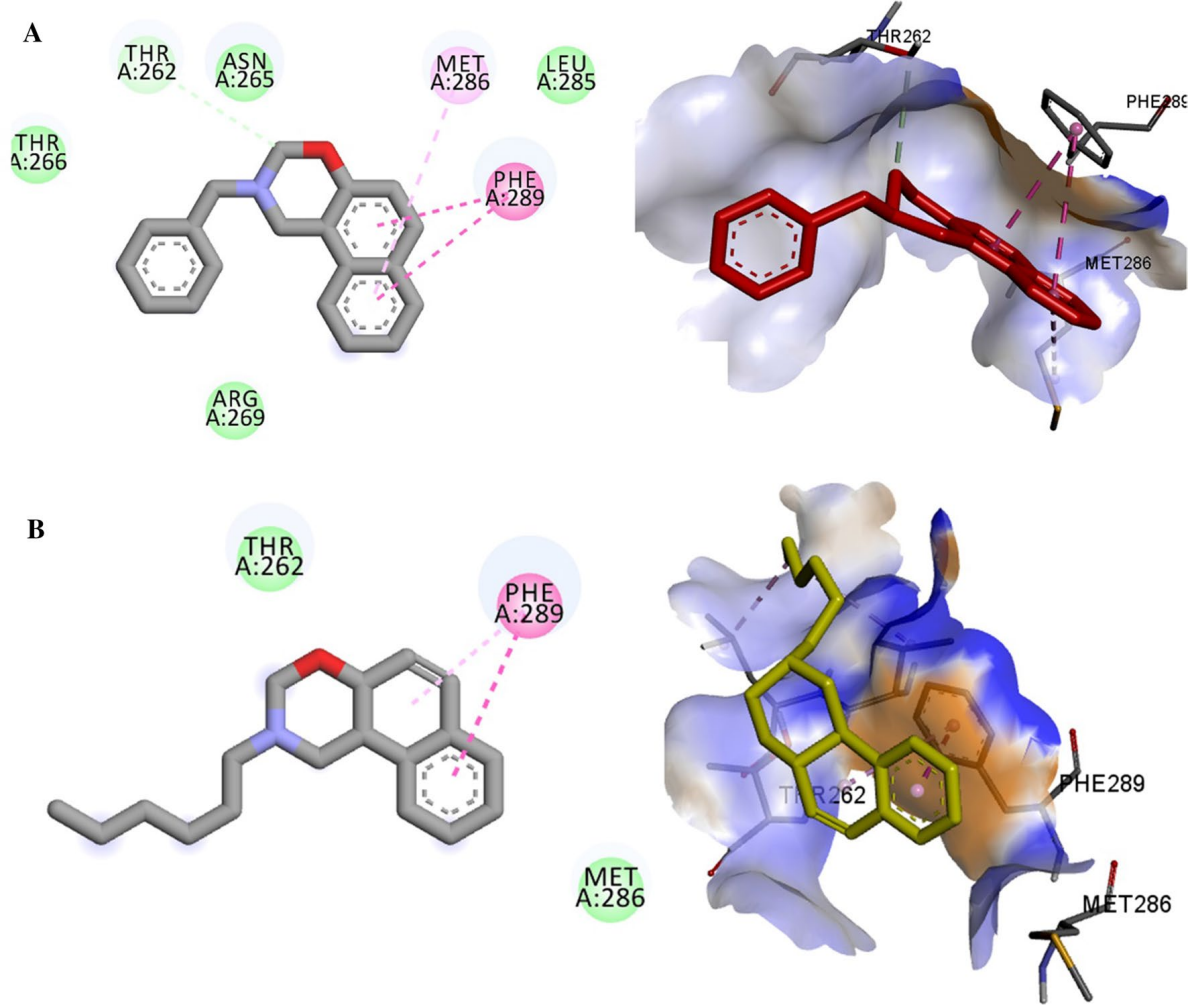
Interactions of ***S***_***2***_ (**A**) and ***S***_***6***_ (**B**) with the residues in the binding site of GABA-A receptor

Molecular docking studies were done to understand the specific binding and mode site of the oxazine derivative to the GABA-A receptor as its proposed target. The results were shown in (Additional file 1: Table S1). The binding mode of the most active (***S***_***10***_, ***S***_***11***_, ***S***_***2***_) and also less active compound (***S***_***6***_) were displayed in Figs. 9 and 10. The full structure of receptor was shown in Figs. 8, 9.

In the case of compound ***S***_***10***_, phenyl moiety of oxazine ring established pi-sulfur interaction with Met 286 residue, moreover, the ethyl group and oxazine core formed alkyl and pi-alkyl (hydrophobic) interaction with Val 290 and Phe 289, respectively. The side chains consisting of amino acid residues Thr 262 and Asn 265 create van der Waals contacts with ***S***_***10***_. On the other hand, compound ***S***_***11***_ as the most potent compound shows the interaction as well as compound ***S***_***10***_. It presented pi-sulfur with Met 286 and pi-alkyl interaction with Phe 289.

As depicted in Fig. 10, the key amino acids in the binding mode of compound ***S***_***2***_ are Met 286 and Phe 289, which interact with the naphthalene moiety by pi-pi and pi-alkyl interactions. Also, some hydrophobic interactions with Thr 266, Thr 262, Asn 265, Leu 285, and Arg 262 are observed. On the other hand, regarding compound ***S***_***6***_ with less anticonvulsant activity, involved in pi–pi interaction with Phe 289, and there is also existed some hydrophobic interactions with residue Thr 262 and Met 286.

### Molecular dynamics simulation and analysis of MD trajectories

MD simulation is a powerful tool that can predict the manners of molecules in biological systems. MD is also an operational method to understand the structural refinements of ligand and the receptor complexes [36, 37]. Molecular dynamics simulation can be accomplished further to study molecular docking results of the ***S***_***10***_ and GABA-A receptor, to investigate their stability and intermolecular interaction profiling with respect to the time [38].

The graphs related to all RMSD, RMSF, number of hydrogen bonds, and radius of gyration (Rg) analysis are widely used in predicting the affinity of ***S***_***10***_ with GABA-A receptor in the active site as compared with the native ligand diazepam.

**Fig. 11:**
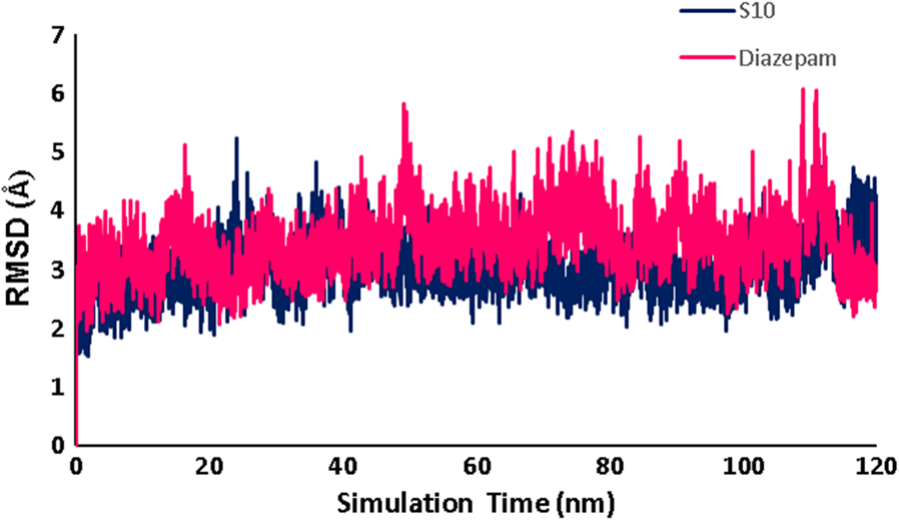
RMSD of the backbone of protein in complex with **S**_**10**_, diazepam at 120 ns MD simulations

RMSD analyses were also investigated to verify the stability of protein and the ligand in each complex. As shown in Fig. 11, the RMSDs of the two systems remain stable and with low fluctuation. The average RMSD of ***S***_***10***_ and native ligand (diazepam) in complex with 6X3X were 3 and 2.7 Å, respectively, during simulation times. As can be seen, two simulated systems showed low RMSD values, reached a plateau form, and showed that the ligands have good stability in the protein's active site. This indicates that, regarding the RMSD analysis, ***S***_***10***_ may show similar performances to the native ligand (diazepam).

RMSF was computed in order to evaluate the effect of the binding of ligand on the protein flexibility and the residual vibration during the simulation time. RMSF analysis was applied to investigate the changing of the flexibility of each amino acid and the residual mobility of protein during MD simulation [39].

**Fig. 12:**
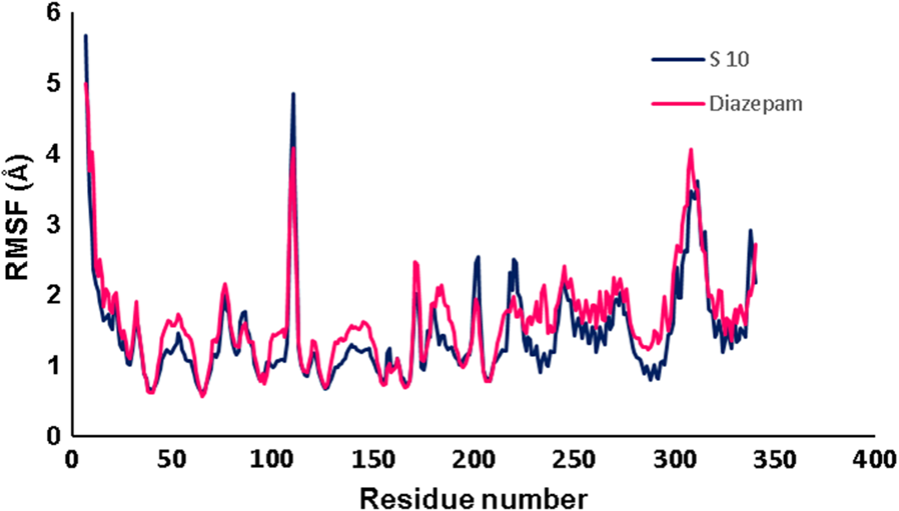
RMSF values of the backbone of protein over the simulation times

The RMSF of the backbone residue of 6X3X was calculated to investigate the fluctuation of amino acid residues. The lowest RMSF values showed the stability, flexibility, and compactness of the protein. The RMSF values of ***S***_***10***_ and diazepam have been shown in Fig. 12. According to the result, RMSF of the protein in complexes with ***S***_***10***_ and diazepam have the same profile and similar distributions. These results indicated that the binding of ***S***_***10***_ made the protein flexible in all areas, same as native co-crystal ligand during 120 ns simulation. The RMSF plot confirmed the absence of changes in the structure of 6X3X through binding of the ***S***_***10***_. According to the inconsiderable changes observed for involved amino acids in the active site, no amino acid residues had a RMSF value > 5 Å. Overall, the critical residues such as Ala37, Thr 58, Pro 78, Gln90, Phe 289, Thr 262, Asn 265, Arg 269, Met 261, Gly 287, Met 286, Phe291, Val290, and Phe330 in the binding pocket are found without any abnormal fluctuation while they are not very much flexible for both of the complexes and they had relatively low RMSF values. These results revealed that the key interactions of the ligand in the binding pocket might maintain stability.

Most of the peaks correspond to the residues 98–117 and 300–325 which they are not involved in the binding pocket of the receptor.

Figure 12 demonstrated the superimposed of before (docking) and after (MD) the structure ***S***_***10***_ and diazepam. Interaction and binding conformation of the two ligands in the GABA-A binding site was confirmed before and after MD simulation.

**Fig. 13:**
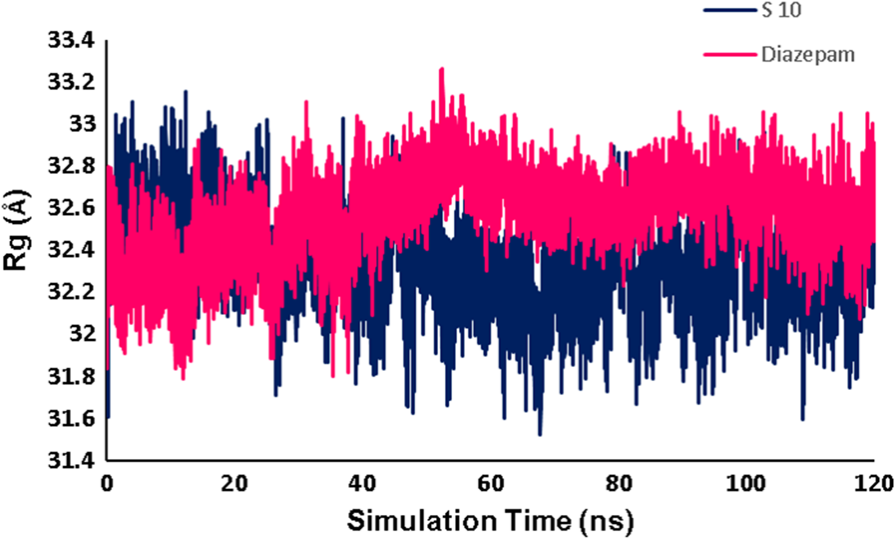
The radius of gyration (Rg) over the simulation time for ***S***_***10***_ and diazepam

The radius of gyration (Rg) of a protein is described as the root mean square distance of each amino acid atom from the core of the protein and reflects its compactness [40]. The plot of the 6X3X radius of gyration during the MD simulations time was demonstrated in Fig. 13. The low values of Rg observed the protein compactness and point to protein stability. According to the Rg plot, based on average Rg, the diazepam and ***S***_***10***_ complexes have the same Rg platform, which was calculated as 30.25 and 30.23 Å, respectively indicating that the stability and compactness of the protein are still maintained by interaction with ***S***_***10***_ compared to native ligand (diazepam). In the case of ***S***_***10***_ complex, the lowest Rg value is observed from 50 to 120 ns, which shows greater rigidness in contrast to the diazepam complexes. It is concluded that ***S***_***10***_ is a suitable inhibitor for the GABA-A target.

**Fig. 14:**
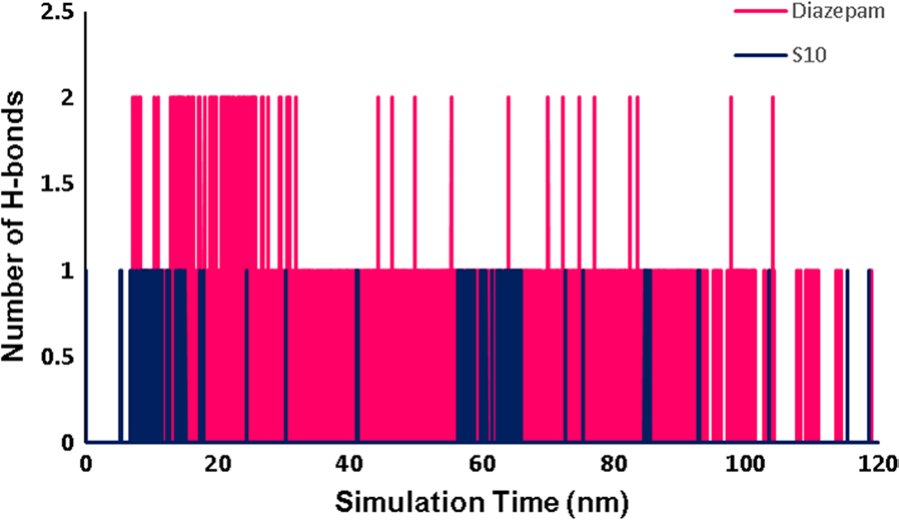
Total number of H-bond count throughout the simulation time of ***S***_***10***_ and diazepam with GABA-A active site

The number of intermolecular hydrogen bonds in the ligand–protein complex are contributed to the conformational stability of the complex. The analysis of H-bond interactions between each ligand and 6X3X was also calculated, and the results are demonstrated in Fig. 14. ***S***_***10***_ showed little interactions with active site residues over the 120 ns simulation compared to the reference molecule, diazepam.

Free binding energies were predicted by the MM-PBSA method available in GROMACS software packages using g_mmpbsa tool. The MM-PBSA method was also applied to evaluate the relative stabilities and molecular interactions of the ligands binding domain with the protein [41, 42].

The formula of binding free energy was as follows:

1 $$\Delta {\text{Gbind }} = {\text{ G complex}}{-}{\text{Gfree protein}}{-}{\text{Gfree ligand}}$$

where G complex, Gfree-protein and Gfree-ligand are the complex energy, the receptor energy and the energy of the unbound ligand, respectively [43].

**Fig. 15:**
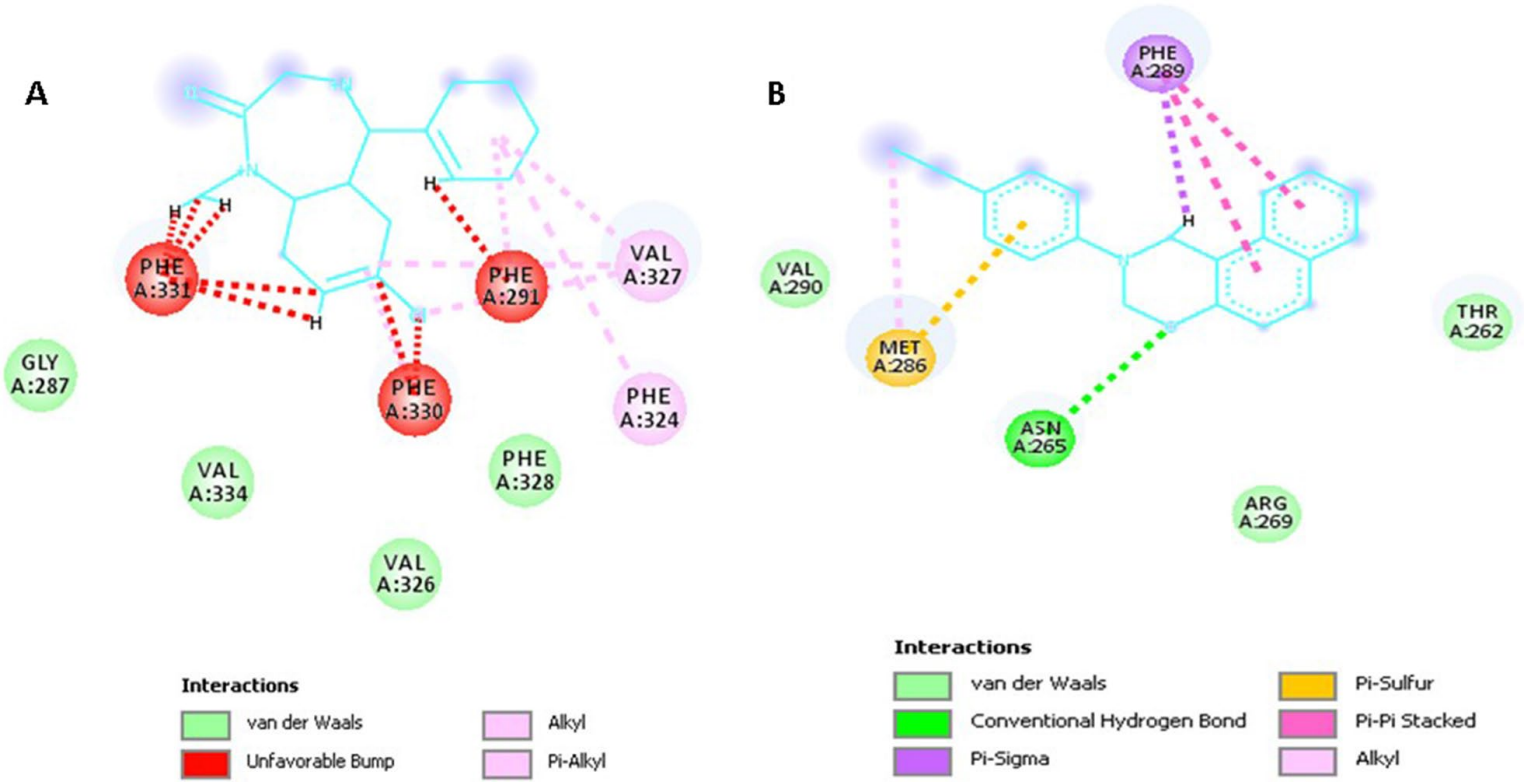
Binding poses of the Diazepam (**A**) and ***S***_***10***_ (**B**) obtained from MD simulations

The calculated energies, including the total binding, Vander walls (VdW) and electrostatic energies (Elec) and SASA from MD trajectories for ***S***_***10***_ and, diazepam are presented in Table 6. According to the results, ***S***_***10***_ has high binding energy in comparison to diazepam and also, forms stable complex with GABA-A receptor (6X3X), which is an agreement with the docking results. Therefore, to recognize the ***S***_***10***_ and diazepam interacting patterns in the active site of receptor after simulation, the proposed binding mode of these compounds generated as 2D interaction plot (Fig. 15). According to the results, ***S***_*10*_ has better binding affinity and more interactions with 6X3X in comparison with diazepam as a reference compound. ***S***_***10***_ gets stabilized by various interactions such as van der walls, conventional hydrogen bonds, pi-sigma, pi-pi, pi-sulfur, and alkyl interactions, while in the case of diazepam, van der walls, alkyl, and pi-alkyl interactions play an important role in receptor binding site. Regarding these interpretations, the higher bonding energy of ***S***_***10***_ was expected.

**Table 6:** Binding free energy components of ***S***_***10***_ and diazepam in complex with 6X3X pdb code GABA-A receptor

System	Energy (Kcal/mol) ± SD				
	VdW^a^	Elec^b^	Polar solvation	SASA^c^	Binding
***S***_***10***_	− 41.486 ± 2.277	− 13.092 ± 3.363	3.451 ± 1.273	− 4.722 ± 0.925	− 37.168 ± 2.541
Diazepam	− 30.098 ± 2.923	− 6.217 ± 3.154	2.631 ± 1.854	− 4.064 ± 0.929	− 27.350 ± 2.798

^a^Vander walls; ^b^Electrostatic energies; ^c^Solvent Accessible Surface Area

## Conclusion

In this study, we develop a magnetically reusable nanocatalyst to synthesize naphto-1,3-oxazine as anticonvulsant agents. Green synthesis, short reaction time, easy to work up, and recoverability of catalyst are the benefit of this method. Furthermore, the preliminary anticonvulsant evaluation was performed in rats by using the ipPTZ test. The results revealed that compounds ***S***_***2***_*, ****S***_***9***_*, ****S***_***10***_, and ***S***_***11***_ significantly increased the seizure threshold. The SAR (Structure Activity Relationship) studies illustrated that compounds in which electron-donating phenyl group directly placed on the oxazine ring could enhance the anticonvulsant effect. Also, the most potent compounds had reasonable physicochemical properties with desired predicted drug-likeness values. Additionally, the molecular docking simulation represented that Thr 262, Asn 265, Met 286, Phe 289, and Val 290 are the key amino acids in the active site and also, there was a desirable correlation between the results of docking studies and anticonvulsant activities. ***S***_***10***_ and diazepam matched up perfectly during the 120 ns of simulation. The results of docking studies of ***S***_***10***_ and diazepam matched up perfectly with MD simulations results. So, we represented a new scaffold that could be further optimized for future development as anticonvulsant agents.

## Supplementary Information

**Additional file 1:** **Figure S1**. The FT-IR spectrum of S1, **Figure S2**. The ^1^H NMR spectrum of S1, **Figure S3**. The FT-IR spectrum of S2, **Figure S4**. The ^1^H NMR spectrum of S2, **Figure S5**. The ^13^C-NMR spectrum of S2, **Figure S6**. The ^13^C-NMR spectrum of S2, **Figure S7**. The FT-IR spectrum of S3, **Figure S8**. The ^1^H NMR spectrum of S3, **Figure S9**. The FT-IR spectrum of S4, **Figure S10**. The ^1^H NMR spectrum of S4, **Figure S11**. The ^13^C-NMR spectrum of S4, **Figure S12**. The FT-IR spectrum of S5, **Figure S13**. The ^1^H NMR spectrum of S5, **Figure S14**. The ^1^H NMR spectrum of S5, **Figure S15**. The FT-IR spectrum of S6, **Figure S16**. The ^1^H NMR spectrum of S6, **Figure S17**. The ^13^C-NMR spectrum of S6, **Figure S18**. The Mass spectrum of S6, **Figure S19**. The FT-IR spectrum of S7, **Figure S20**. The ^1^H NMR spectrum of S7, **Figure S21**. The FT-IR spectrum of S8, Figure S22. The ^1^H NMR spectrum of S8, **Figure S23**. The ^13^C-NMR spectrum of S8, **Figure S24**. The Mass spectrum of S8, **Figure S25**. The FT-IR spectrum of S9, **Figure S26**. The ^1^H NMR spectrum of S9, **Figure S27**. The ^13^CNMR spectrum of S9, **Figure S28**. The Mass spectrum of S9, **Figure S29**. The FT-IR spectrum of S10, **Figure S30**. The ^1^H NMR spectrum of S10, **Figure S31**. The FT-IR spectrum of S11, **Figure S32**. The ^1^H NMR spectrum of S11, **Figure S33**. EDS analysis of the GO-Fe_3_O_4_-Ti_(IV)_, **Figure S34**. VSM plot of pure Fe_3_O_4_, and GO-Fe_3_O_4_-Ti_(IV)_, **Table S1**. The bonding energies (kcal/mol) of the tested compounds on GABA-A using AutoDock Vina.

## Data Availability

The data sets used and analyzed during the current study are available from the corresponding author on reasonable request. We have presented all data in the form of Tables and Figure. The PDB code (6X3X) was retrieved from protein data bank (www.rcsb.org). https://www.rcsb.org/structure/6X3X.

## Abbreviations

PTZ: Pentylenetetrazole
CNS: Central nervous system
GABA: γ-Aminobutyric acid
MD: Molecular dynamic
SAR: Structure Activity Relationship
ipPTZ: Intraperitoneal pentylenetetrazole test
ADME: Adsorption, Distribution, Metabolism and Discretion
Rg: Radius of gyration
RMSD: Root Mean Square Deviation
GO: Graphene oxide
di: Deionized water
XRD: X-Ray diffraction analysis
EDS: Energy-dispersive X-Ray spectroscopy
