# Supplementary material for: Efficient synthesis of 1,3-naphtoxazine derivatives using reusable magnetic catalyst (GO-Fe3O4–Ti(IV)): anticonvulsant evaluation and computational studies
Source: BMC Chem. 2022 Jun 10;16(1):44. doi: 10.1186/s13065-022-00836-8 (PMC9188075; doi:10.1186/s13065-022-00836-8)
Supplement: Supplementary file 1 — Additional file 1: Figure S1. The FT-IR spectrum of S1, Figure S2. The 1H NMR spectrum of S1, Figure S3. The FT-IR spectrum of S2, Figure S4. The 1H NMR spectrum of S2, Figure S5. The 13C-NMR spectrum of S2, Figure S6. The 13C-NMR spectrum of S2, Figure S7. The FT-IR spectrum of S3, Figure S8. The 1H NMR spectrum of S3, Figure S9. The FT-IR spectrum of S4, Figure S10. The 1H NMR spectrum of S4, Figure S11. The 13C-NMR spectrum of S4, Figure S12. The FT-IR spectrum of S5, Figure S13. The 1H NMR spectrum of S5, Figure S14. The 1H NMR spectrum of S5, Figure S15. The FT-IR spectrum of S6, Figure S16. The 1H NMR spectrum of S6, Figure S17. The 13C-NMR spectrum of S6, Figure S18. The Mass spectrum of S6, Figure S19. The FT-IR spectrum of S7, Figure S20. The 1H NMR spectrum of S7, Figure S21. The FT-IR spectrum of S8, Figure S22. The 1H NMR spectrum of S8, Figure S23. The 13C-NMR spectrum of S8, Figure S24. The Mass spectrum of S8, Figure S25. The FT-IR spectrum of S9, Figure S26. The 1H NMR spectrum of S9, Figure S27. The 13CNMR spectrum of S9, Figure S28. The Mass spectrum of S9, Figure S29. The FT-IR spectrum of S10, Figure S30. The 1H NMR spectrum of S10, Figure S31. The FT-IR spectrum of S11, Figure S32. The 1H NMR spectrum of S11, Figure S33. EDS analysis of the GO-Fe3O4-Ti(IV), Figure S34. VSM plot of pure Fe3O4, and GO-Fe3O4-Ti(IV), Table S1. The bonding energies (kcal/mol) of the tested compounds on GABA-A using AutoDock Vina. [file 13065_2022_836_MOESM1_ESM.docx]

**Supporting Information**

**Efficient Synthesis of 1,3-Naphtoxazine Derivatives Using Reusable Magnetic Catalyst (GO-Fe_3_O_4_–Ti^IV^) as Anticonvulsant Agents: Biological Evaluation and Computational Studies**

Soghra Khabnadideh^1^, Aida solhjoo^2^, Reza Heidari^1^, Leila Amiri Zirtol^1^, Amirhossein Sakhteman^2,4^, Elaheh Babaei^3^, Samaneh Rahimi^2^, Leila Emami^1^^[[1]](#footnote-1)^*

*^1^Pharmaceutical Sciences Research Center, Shiraz University of Medical Sciences, Shiraz, I.R.Iran*

*^2^Department of Medicinal Chemistry, Faculty of Pharmacy, Shiraz University of Medical Sciences, Shiraz, I.R. Iran.*

*^3^Department of Chemistry, College of Science, Yazd University, Yazd.*

*^4^Institute of Biomedicine, University of Eastern Finland, Kuopio, Finland. Electronic*

**Figure S1**. The FT-IR spectrum of **S1**

**Figure S2**. The ^1^H NMR spectrum of **S1**

**Figure S3**. The FT-IR spectrum of **S2**

**Figure S4**. The ^1^H NMR spectrum of **S2**

**Figure S5**. The ^13^C-NMR spectrum of **S2**

**Figure S6**. The ^13^C-NMR spectrum of **S2**

**Figure S7**. The FT-IR spectrum of **S3**

**Figure S8**. The ^1^H NMR spectrum of **S3**

**Figure S9**. The FT-IR spectrum of **S4**

**Figure S10**. The ^1^H NMR spectrum of **S4**

**Figure S11**. The ^13^C-NMR spectrum of **S4**

**Figure S12**. The FT-IR spectrum of **S5**

**Figure S13**. The ^1^H NMR spectrum of of **S5**

**Figure S14**. The ^1^H NMR spectrum of **S5**

**Figure S15**. The FT-IR spectrum of **S6**

**Figure S16**. The ^1^H NMR spectrum of **S6**

**Figure S17**. The ^13^C-NMR spectrum of **S6**


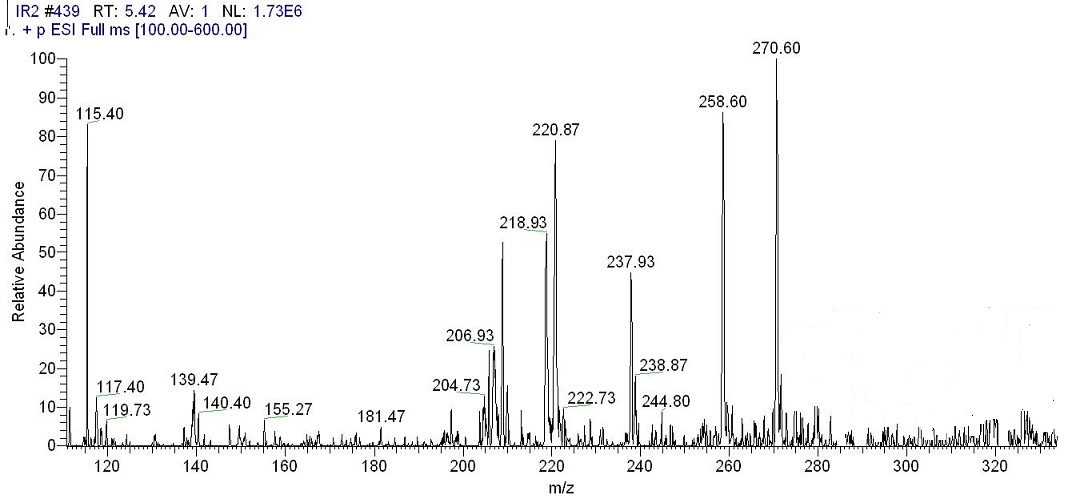


**Figure S18**. The Mass spectrum of **S6**

**Figure S19**. The FT-IR spectrum of **S7**

**Figure S20**. The ^1^H NMR spectrum of **S7**

**Figure S21**. The FT-IR spectrum of **S8**

**Figure S22**. The ^1^H NMR spectrum of **S8**

**Figure S23**. The ^13^C-NMR spectrum of **S8**

**
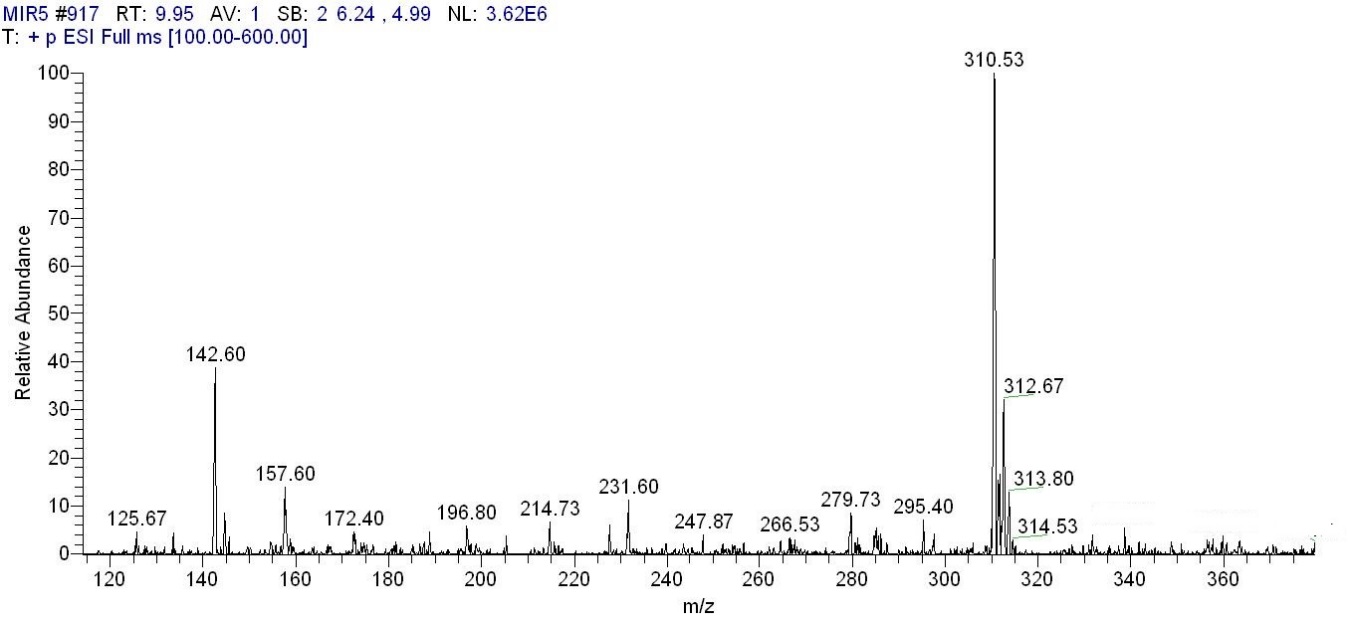
**

**Figure S24**. The Mass spectrum of **S8**

**Figure S25**. The FT-IR spectrum of **S9**

**Figure S26**. The ^1^H NMR spectrum of **S9**

**Figure S27**. The ^13^C-NMR spectrum of **S9**


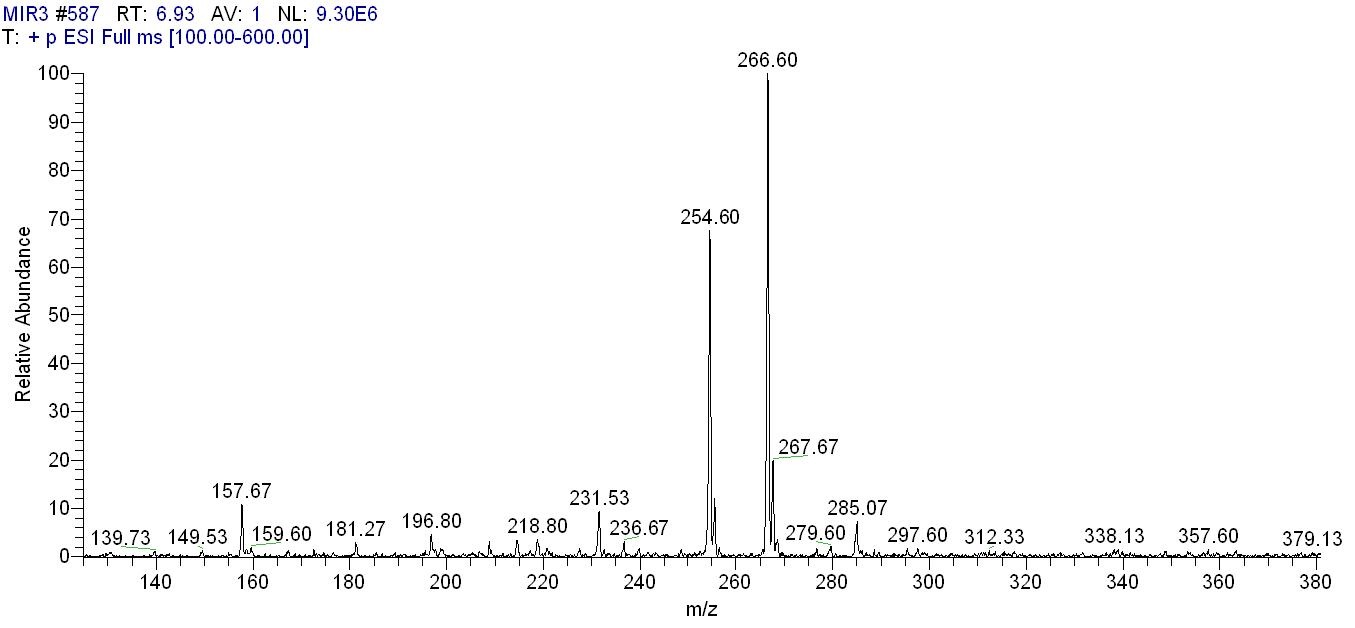


**Figure S28**. The Mass spectrum of **S9**

**Figure S29**. The FT-IR spectrum of **S10**

**Figure S30**. The ^1^H NMR spectrum of **S10**

**Figure S31**. The FT-IR spectrum of **S11**

**Figure S32**. The ^1^H NMR spectrum of **S11**


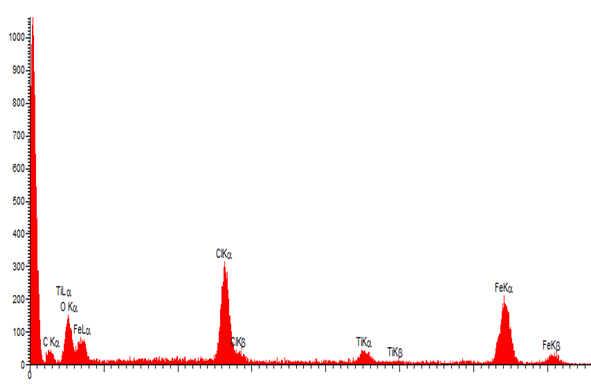
 **Figure S33**. EDS analysis of the GO-Fe3O4-Ti ^(IV)^


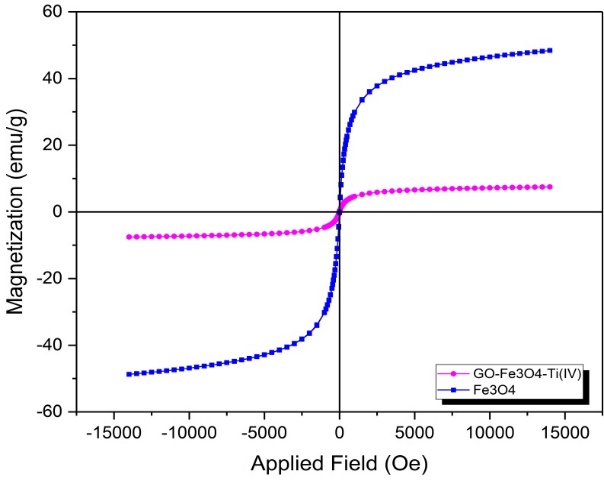


**Figure S34**. VSM plot of pure Fe_3_O_4_, and GO-Fe_3_O_4_-Ti ^(IV^

**Table S1.** The bonding energies (kcal/mol) of the tested compounds on GABA-A using AutoDock Vina

| Entry | ***S_1_*** | ***S_2_*** | ***S_3_*** | ***S_4_*** | ***S_5_*** | ***S_6_*** | ***S_7_*** | ***S_8_*** | ***S_9_*** | ***S_10_*** | ***S_11_*** | CO(crystal ligand) |
| --- | --- | --- | --- | --- | --- | --- | --- | --- | --- | --- | --- | --- |
| 6X3X | -8.1 | -8.2 | -8 | -8.1 | -8 | -7.3 | -8.2 | -8.3 | -8.8 | -9 | -8.9 | -8.7 (Diazpam) |

1. *Corresponding author: Leila Emami, Tel: +98 -71-32424127-8; Fax: +98-71-32424126; E-mail: emamil@sums.ac.ir [↑](#footnote-ref-1)
